# Assessing the magnitude and uncertainties of the burden of selected diseases attributable to extreme heat and extreme precipitation under a climate change scenario in Michigan for the period 2041–2070

**DOI:** 10.1186/s12940-019-0483-5

**Published:** 2019-04-27

**Authors:** Carina J. Gronlund, Lorraine Cameron, Claire Shea, Marie S. O’Neill

**Affiliations:** 1 Center for Social Epidemiology and Population Health, University of Michigan School of Public Health, 1415 Washington Heights, Ann Arbor, MI 48109-2029 USA; 2 Michigan Climate and Health Adaptation Program, Division of Environmental Health, Michigan Department of Health and Human Services, 333 S. Grand Ave, Lansing, MI 48909 USA; 3 Departments of Epidemiology and Environmental Health Sciences, University of Michigan School of Public Health, 1415 Washington Heights, Ann Arbor, MI 48109-2029 USA

**Keywords:** Climate change, Morbidity, Mortality, Emergency department, Hospitalization, Extreme heat, Extreme precipitation

## Abstract

**Background:**

Extreme heat (EH) and extreme precipitation (EP) events are expected to increase with climate change in many parts of the world. Characterizing the potential future morbidity and mortality burden of EH and EP and associated costs, as well as uncertainties in the estimates, can identify areas for public health intervention and inform adaptation strategies. We demonstrate a burden of disease and uncertainty assessment using data from Michigan, USA, and provide approaches for deriving these estimates for locations lacking certain data inputs.

**Methods:**

Case-crossover analysis adapted from previous Michigan-specific modeling was used to characterize the historical EH-mortality relationship by county poverty rate and age group. Historical EH-associated hospitalization and emergency room visit risks from the literature were adapted to Michigan. In the U.S. Environmental Protection Agency’s BenMAP software, we used a novel approach, with multiple spatially-varying exposures, to estimate all non-accidental mortality and morbidity occurring on EH days (EH days; days where maximum temperature 32.2–35 C or > 35 C) and EP days. We did so for two time periods: the “historical” period (1971–2000), and the “projected” period (2041–2070), by county.

**Results:**

The rate of all non-accidental mortality associated with EH days increased from 0.46/100,000 persons historically to 2.9/100,000 in the projected period, for 240 EH-attributable deaths annually. EH-associated ED visits increased from 12/100,000 persons to 68/100,000 persons, for 7800 EH-attributable emergency department visits. EP-associated ED visits increased minimally from 1.7 to 1.9/100,000 persons. Mortality and morbidity were highest among those aged 65+ (91% of all deaths). Projected health costs are dominated by EH-associated mortality ($280 million) and EH-associated emergency department visits ($14 million). A variety of sources contribute to a moderate-to-high degree of uncertainty around the point estimates, including uncertainty in the magnitude of climate change, population composition, baseline health rates, and exposure-response estimates.

**Conclusions:**

The approach applied here showed that health burden due to climate may significantly rise for all Michigan counties by midcentury. The costs to health care and uncertainties in the estimates, given the potential for substantial attributable burden, provide additional information to guide adaptation measures for EH and EP.

**Electronic supplementary material:**

The online version of this article (10.1186/s12940-019-0483-5) contains supplementary material, which is available to authorized users.

## Background

In this changing climate, state and local health departments in the USA and elsewhere are considering how best to protect the public’s health. Approaches to estimate future burden of disease of climate-related health outcomes and the associated costs and uncertainties can guide planning and fiscal policy. Detailed examples of how these approaches can be applied in a specific place--in this case, Michigan, USA--offer a template for their use elsewhere.

During a strategic planning initiative in 2010, the Michigan Department of Health and Human Services Climate and Health Adaptation Program identified specific health outcomes as priority concerns when considering the impacts from climate change in Michigan [1], including but not limited to heat-related illnesses and waterborne diseases. These effects were chosen based on evidence that Michigan-specific climate change effects include increases in EH events and increases in extreme precipitation (EP) events and concomitant flooding events [2, 3].

Following this scoping phase, we estimated the present and future burden of disease associated with these health outcomes. We followed the U.S. Centers for Disease Control and Prevention technical guidance for health departments for projecting climate-related disease burden [4] by, 1) developing a causal pathway linking exposures/environmental hazards to health outcomes, 2) using ensemble projections from global climate models (GCMs) to identify how the exposures/environmental hazards may change in intensity and duration in the future, 3) establishing the historical disease burdens of the health outcomes in our populations, 4) assessing the historical exposure-outcome associations, 5) estimating the health burdens historically and in the projected climate, and then 6) evaluating the uncertainty inherent in the derivation of these different estimates.. The results will help prioritize county level measures to better protect public health in a changing climate. We also present our methods as a model to other states and municipalities, including methods for deriving baseline health data and exposure-response estimates when these are lacking.

## Methods

**Fig. 1:**
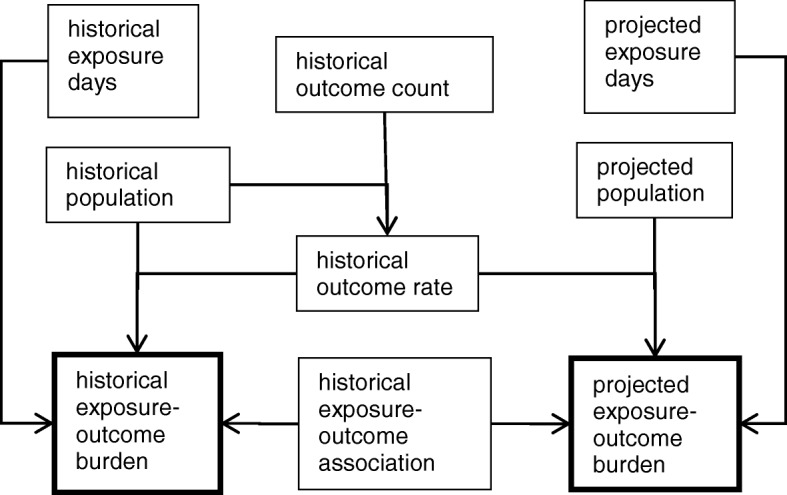
Steps in burden-of-disease estimates for outcomes of mortality, hospitalization and emergency department (ED) visits for the extreme heat (EH) exposure and the outcome of gastrointestinal (GI) ED visits for the extreme precipitation (EP) exposure. See Table 1 for data sources

An overview of the methods is provided below and summarized in Fig. 1 and Table 1. Additional details sufficient to replicate the analyses are provided in Additional file 1: Appendix 1.

**Table 1:** Source of data for each step (Fig. 1) in the burden of disease calculation and years of data used

Step	Data Source	Historical	Projected
Extreme heat (EH) days	Maurer 1/8-degree gridded daily maximum temperature observations [6]	1971–2000	
Extreme heat (EH) days	Multi-model ensemble^b^ of statistically-downscaled 1/8-degree dailydata sets from the North American Regional Climate Change Assessment Program [3]		2041–2070
Extreme precipitation (EP) days	Multi-model ensemble^b^ of statistically-downscaled 1/8-degree daily projections [3, 5, 14, 15]	1971–2000^a^	2041–2070
Population	U.S. Census [16]	1971–2000	
Population	Woods & Poole economic forecasting model [17]		2050
Population	EPA’s Integrated Climate and Land-Use Scenarios (ICLUS) project for the A2 scenario [16, 103]		2050
All-natural-cause mortality	Centers for Disease Control (CDC), National Center for Health Statistics (NCHS) [17]	2004–2006	
Renal/respiratory/heat hospitalizations, ages 65+	Medicare MedPAR billing records [20]	1990–2006	
Renal hospitalizations, ages 0–64	Michigan Inpatient Database [18]	2000–2009	
All-natural-cause and gastrointestinal emergency department (ED) visits	Healthcare Cost and Utilization Project, Agency for Healthcare Research and Quality [21]	2007	
EH-mortality association	case-crossover analysis, see Methods		
EH-renal/respiratory/heat hospitalization association	Ogbomo et al. [18]	2000–2009	
EH-renal hospitalization association	Gronlund et al. [20]	1990–2006	
EH-all-natural-cause ED visit association	Kingsley et al. [25]	1999–2011	
EP-GI ED visit association	Jagai et al. [27]	2003–2007	

^a^No additional data source needed; by definition, 2% of days in the historical period are EP days

^b^Derived from the following six Climate Model Intercomparison Project Phase 3 global climate models (GCMs): cgcm3_t47, cgcm3_t63, cnrm, echam5, gfdl_2.1, pcm

### Review of causal pathways

For the priority health outcomes, we reviewed the literature concerning EH-associated mortality, EH-associated hospitalizations, EH-associated emergency department (ED) visits, EP-associated gastrointestinal (GI) illness, and EP-associated respiratory illness. We focused specifically on studies including Michigan residents, and in the absence of such studies, we selected studies of U.S. populations in climates similar to that of Michigan. Based on the availability of studies and their quality, we chose whether to perform quantitative estimates of disease burden for a given exposure-outcome association or to assess the burden qualitatively.

### Multi-GCM ensemble projections

#### EH

The mean annual number of days with maximum temperatures above 32.2 °C and above 35 °C, or EH days, were obtained for two time periods: 1971–2000 (historical) and 2041–2070 (projected) from the Great Lakes Integrated Sciences + Assessments Center [5]. These were derived under the Intergovernmental Panel on Climate Change A2 scenario, which is one of the more dire scenarios and assumes greenhouse gas concentrations will continue to increase throughout the twenty-first century [2]. The historical EH data are the Maurer 1/8-degree gridded daily maximum temperature observations [6]. The projections were statistically-downscaled data sets from the North American Regional Climate Change Assessment Program [3] derived from Climate Model Intercomparison Project Phase 3 GCMs. Estimates of EH days were provided at a 1/8° grid resolution and were aggregated to match the spatial resolution of the health data (see Additional file 1: Appendix 2 for aggregation details).

No universal definition of EH exists, and the thresholds of 32.2 °C and 35 °C were chosen for two reasons. Firstly, these correspond to round Fahrenheit temperatures of 90 °F and 95 °F, and extreme caution is advised by the local Detroit National Weather Service office at temperatures over 90 °F [7]. Although these were well above the minimum-mortality-temperature thresholds of 21–24 °C identified in Gasparrini et al. [8] for cities in Michigan, the 32.2 °C threshold corresponds roughly to a Detroit heat-mortality threshold in Gronlund et al. [9], which used more recent, albeit more spatially constrained, mortality time series. Secondly, time series and case-crossover study designs, on which our estimates are based, can account for mortality displacements of 1–3 weeks, thereby subtracting out the mortality attributable to heat that was among very frail individuals who would have died within 1–3 weeks anyway. Beyond 3 weeks, we are not aware of research quantifying mortality displacement specific to cities in Michigan, although the extent of mortality displacement varies widely between countries [10]. The literature is inconclusive, but there is some suspicion that this mortality displacement is reduced at very high temperatures [11–13]. Therefore, in using high EH thresholds, we have greater confidence in assigning years, rather than weeks, of life lost to EH.

#### EP

Based on an ensemble of downscaled daily climate projections [3, 5, 14, 15] (Table 1) for the A2 scenario, EP events (the heaviest 2% of precipitation events in a given area) are projected to increase in Michigan between an additional 0.5 days to over 2.0 days per year.

### Historical disease burden of the health outcomes

#### Mortality rates

The U.S. Environmental Protection Agency’s (EPA) Environmental Benefits Mapping and Analysis Program (BenMAP) is a free, geographic information system (GIS)-based software that calculates and maps the health impacts associated with changes in air quality or temperature [16]. The BenMAP software contains past and present age-, cause-, and county-specific mortality rates derived from National Center for Health Statistics and U.S. Census data [17].

#### Hospitalization rates

BenMAP does not include information on renal or heat-related (i.e., specific effects of heat such as heat exhaustion and heat stroke) hospitalization rates. Therefore, annual warm season (May–September) renal hospitalization rates were estimated from a Michigan study of EH and hospitalization for individuals 0–64 years of age [18] in conjunction with U.S. Census data [19]. For individuals 65 years and older, hospitalizations for renal, respiratory and heat causes were derived from a study of EH and hospitalization, which included Michigan [20].

#### ED rates

Because Michigan does not provide state or discharge-level data on ED visits, Michigan-specific ED visit rates for heat-related and non-accidental causes were estimated using Healthcare Cost and Utilization Project (HCUP) data [21] from the Midwest region and applied to all Michigan counties. See Section 6 for Results regarding the variation in ED visit rates among states in the Midwest and Additional file 1: Appendix 3 for detailed Methods and Results.

#### Population counts

Historical population data from the 2000 U.S. Census [16] were available in BenMAP. We used 2050 population projections from EPA’s Integrated Climate and Land-Use Scenarios (ICLUS) project for the A2 scenario. ICLUS population projections are based on the 2000–2005 U.S. Census population, fertility, and mortality rates by age, sex, and race to project county-specific populations out to 2100 [22]. For the A2 scenario, the resulting ICLUS population includes an assumption of higher fertility rates. As a sensitivity analysis, we used BenMAP’s pre-loaded Woods & Poole 2050 projections.

### Historical exposure-outcome associations

#### EH-mortality association

Land cover by heat-retaining surfaces, minority racial status, and low socioeconomic status have been found in previous research to increase vulnerability to EH [23]. To estimate the EH-mortality association, we performed a new epidemiologic analysis based on a recent Michigan-specific analysis by Gronlund et al. [9] using Michigan mortality records, airport temperature data, and ZIP-code level estimates of green space, percent of residents of black race, percent of individuals 65 years and older and living alone, and percent below the poverty level. Based on the previous analysis suggesting a great reduction or absence of an EH-mortality association beyond 3 days after the EH exposure in this region, we restricted the analysis to the day of through 3 days following the EH exposure. We updated the analysis to use an EH definition that precisely matched that in the available climate projections and to include all ages. Attributable fractions (AFs), or the fraction of deaths attributable to 1 day of EH, were calculated for each ZIP code*,* age group and EH threshold (32.2 or 35 °C) based on the derived risk ratios (RRs). Race was not found to be a significant modifier of the EH-mortality association when land cover and socioeconomic characteristics were accounted for. Although green space was found to significantly modify the EH-mortality association, it was not included in the AF estimate due to the fact that green space was a variable used in estimating the number of historical and projected EH days.

#### EH-hospitalization association

Strong associations between EH and renal-cause hospitalizations have been found in several U.S. studies [24], as has significant effect modification by white vs. black race among individuals 65 and older in the U.S. [20]. From a Michigan-specific study by Ogbomo et al. [18], we estimated an AF for individuals under 65 years of age at a same-day EH threshold of 32.2 °C. Renal effects for 2-, 3-, and 4-day long EH events were similar to same-day EH effects, and Ogbomo et al. only estimated effects by age group for same-day EH events. For individuals 65 years of age and older, we used the air conditioning prevalence region 2 (which included Michigan) ZIP-code specific AFs for six-day cumulative effects of EH at 32.2 °C from the U.S. study of effect modification of renal, respiratory and heat-related hospitalizations by Gronlund et al. [20].

#### EH-ED visit association

Exposure-response functions were derived from a study examining the effects of heat on morbidity and mortality by Kingsley et al. in Rhode Island [25], a state with a climate similar to that of Michigan’s, i.e., cold and lacking a dry season and hot summers (Dfa, Dfb) in the Köppen climate classification system [26]. AFs for all-natural-cause ED visits for EH at 32.2 °C and 35 °C were estimated for the 0–18 age group and the 65 and older age group based on effect estimates of same-day maximum temperature and all-natural cause visits. In contrast to the mortality and hospitalization studies, Kingsley et al. did not estimate effects of temperature on ED visits on days following the EH exposure °s. The association between same-day maximum temperature and all-natural-cause ED visits was not significant in the 18–64 age group. However, associations between *heat-related* ED visits and warm temperatures were strong in this age group; so heat-related AFs for 32.2 °C and 35 °C were estimated for 18–64 year-olds.

#### EP-ED visit association

The exposure-response function was derived from a study by Jagai et al. examining the association between EP and GI illness in areas with and without combined-sewer overflows (CSOs) in Massachusetts [27]. The study was chosen due to its similar climate [26] as well as stratification by region according to the impact of CSOs: regions where the CSOs impacted drinking water sources, regions where the CSOs impacted recreational waters, and regions without CSOs. Such stratification was recommended in a recent review of 24 studies of the association between EP and waterborne infections [28].

### 5. Historical and projected EH- and EP-attributable health burdens

#### Attributable burden counts

For each time period *p* (historical vs. projected)*,* county *c*, age group *a,* race category *r,* and threshold *t* (32.2–34.9 °C or ≥ 35 °C for EH and historical 2% heaviest rainfall amount for EP)*,* we calculated the attributable fraction days (*AFD*_*cartp*_) as the product of the *AF*_*cart*_ and the number of EH or EP days in that time period (*EHD*_*p*_). *AFD*_*carp*_ was then calculated as the sum of the two *AFD*_*cartp*_ values, one from each threshold. For mortality and hospitalization estimates, we first calculated AFD by ZIP code *z* instead of county, and then we calculated *AFD*_*carp*_ as the weighted average of the *AFD*_*zarp*_ in that county, where the weights were the number of cases in that ZIP code and age-race group (Additional file 1: Appendix 4). For all health outcomes, the burden-of-disease function was then defined in BenMAP as

$$ {BOD}_{carp}={AFD}_{carp}\times {IR}_{carp}\times {POP}_{carp}\times \mathrm{C} $$

where *BOD*_*carp*_ was the burden of disease due to EH or EP in county *c* in age group *a* and race group *r* in time period *p* (historical or projected), *IR* was the county-level (or state-level for ED visits) *daily* incidence rate, *POP* was the population, and *C* scaled the incidence rate (for EH estimates only) from a daily annual to daily summer level, given reduced incidence in the summer vs. winter (0.66 for emergency-department visits and 0.39 for mortality). Of note, these BOD estimates assume that the entire population was exposed, or in a region experiencing that number of EH or EP days. The *BOD*_*carp*_ estimates were then summed across age-race groups to generate the county and time-period-specific estimates. County estimates were summed to generate the statewide estimates. For EP, because neither our exposures nor our exposure-response functions varied geographically, we did not use BenMAP, and *BOD* was estimated for the state as a whole for residents with drinking water from surface water sources.

#### Attributable monetary costs

To estimate the monetary statewide costs of the attributable burdens of each disease, we multiplied the period-specific statewide BOD estimates by the per-incident cost of death, renal hospitalization among whites and non-whites, non-accidental ED visit, or GI illness ED visit. The mortality incident cost was the dollars per quality-adjusted life-year (QALY) estimated in an incremental cost-effectiveness ratio analysis of current dialysis practices relative to less-costly alternatives [29]. Dialysis is often used as a benchmark of an amount that is justifiably paid to improve quality-adjusted life years and therefore a reasonable proxy of the value of a year of life [29]. To estimate the cost of heat-associated hospitalization associated with EH, we used a cost of $5400, with additional costs of $1500 for individuals 65–77 and $1600 for individuals 78 and older from a study of hospitalization costs due to heat-related illness [30]. For respiratory and renal hospitalization costs, we used Healthcare Cost and Utilization Project 2014 Michigan data by age group [21]. To estimate ED visit costs, we calculated the median costs of non-accidental and gastrointestinal ED visits in the U.S. from the 2015 Medical Expenditure Panel Survey [31]. All cost results are given in 2010–2015 U.S. dollars, with no adjustment for inflation in the projected period.

### Uncertainties

Rather than attempt to assign precise quantitative ranges to the burden-of-disease inputs, we took a qualitative approach. To each source of uncertainty, we assigned values of “low,” “moderate,” or “high,” defined as approximate ranges around the point estimates of ±49% for low, − 99% to − 50% or + 50% to + 199% for moderate, and − 100% or ≥ + 200% for high. This simple scale represents the idea that ranges of effects within half-as-much above or below the point estimate reflect low uncertainty, ranges that include null or protective effects (more than 100% below) or effects more than three times as high reflect high uncertainty, and other ranges reflect moderate uncertainty. We drew on quantitative information when assigning these values, but we discuss additional uncertainties where present.

In order to evaluate uncertainty in population growth and distribution patterns, BOD estimates generated in BenMAP using the 2050 ICLUS population data for the A2 scenario were compared to the results derived using 2050 Woods & Poole data [17]. The Woods & Poole population projections are based on an economic forecasting model [32] while the ICLUS project used a demographic model with migration rates consistent with the IPCC’s A2 scenario [33].

In generating the EH and EP day projections, Hayhoe et al. [15] did a bias analysis of each of the GCMs used to generate the “ensemble” mean projection for the US. In short, this analysis was accomplished by comparing the EH or EP days for the period 1960–1999 predicted by the models to those that actually occurred in that period. We reviewed the bias analyses relevant to the GCMs used to generate our historical and projected number of EH and EP days. We regard these as rough estimates of the bias in generating the A2 projection.

We also evaluated uncertainty in the exposure-response association and the baseline estimates of the health effects themselves, considering differences between studies of similar outcomes and changes in the health effect rates over time. To evaluate the uncertainties in the cost estimate of mortality, we used the estimated 1st and 99th percentiles of the dollar-per-QALY estimate [29]. For hospitalizations, we considered the 95% confidence intervals around estimated heat-related illness hospitalization costs [30]. For ED visits, we examined the first and third quartiles of the non-accidental and infectious intestinal illness visit costs estimated from the 2015 Medical Expenditure Panel Survey [31]. Using estimates of renal hospitalization counts [21] and annual population estimates [34], we compared age-adjusted renal hospitalization rates in 2001–2003 and 2012–2014. We also considered trends in ED visit rates from 2006 to 2011 [35].

## Results

### Review of causal pathways

#### EH

As part of the Climate and Health Profile Report, several direct and indirect health impacts related to EH and EP were identified [1]. Pathways by which EH affects health are direct and have been reviewed elsewhere [24, 36–38]. Briefly, mortality due to non-accidental causes was chosen as an indicator of disease burden for this analysis due to its known direct and immediate association with EH events [36, 37]. Non-accidental-cause ED visits and renal, respiratory and heat-related hospitalizations were chosen to best reflect the impact of EH on a wide variety of chronic diseases. Previous studies have shown associations between EH events and non-accidental-cause ED visits [38] and between EH and renal, respiratory and heat hospitalizations [24, 38]. Therefore, we included non-accidental cause mortality; renal, respiratory, and heat-related hospitalizations; and non-accidental ED visits in our EH burden of disease estimates, using studies that included Michigan and provided region-specific results, where possible, and from similar climates otherwise.

#### EP

Multiple pathways between EP and health may exist, although we did not find sufficient quantitative estimates of EP and health for the majority of these pathways to sufficiently characterize the health effects in our quantitative burden of disease estimates. Therefore, we describe these pathways in detail below. EP leads to contamination of surface water by increasing turbidity, by increasing the chances of harmful algal blooms, which are fed by agricultural run-off, and by prompting combined sewer overflows (CSOs). EP may also contaminate surface water when EP leads to flooding and flood waters wash contaminants into the surface water body.

EP events have been found to be associated with GI illness in countries outside the U.S. with inadequate treatment of public drinking water [39, 40]. Even in the U.S. where public water supplies are treated, a small proportion of illness has been attributed to waterborne disease [41]. These waterborne infections are caused by a variety of viruses, bacteria and protozoa. Though rare in Canada and the U.S., EP has been associated with waterborne disease outbreaks in recent decades: a *Cryptosporidium* outbreak in Milwaukee in 1993 and an *Escherichia coli* outbreak in Walkerton, Ontario in 2000 [42].

In addition to GI illnesses, a small number of studies have found an association between precipitation and the respiratory pathogen *Legionella*, or Legionnaires’ disease in its most severe form, which thrives in warm water [43, 44]. In a study of *legionellosis* incidence in five Mid-Atlantic states from 1990 to 2003, Hicks et al. [45] found both monthly temperature (another meteorological variable predicted to increase with climate change) and rainfall to be associated with *legionellosis*. Specifically, Hicks et al. found a 2.6% increased risk of *legionellosis* with each 1-cm increase in rainfall. A study in Switzerland did not find associations between precipitation and *legionellosis*, although the researchers did find associations of *legionellosis* with temperature and water vapor pressure [46]. A case-crossover study of 240 *Legionella* cases in the Philadelphia area from 1995 to 2003 also did not provide evidence of an association between precipitation and *legionellosis* when controlling for other meteorologic factors, but the researchers did find associations between *Legionella* and relative humidity, with RRs of 3.93 (95% Confidence Interval: 2.18–7.09) and 3.59 (95% CI: 2.06–6.28) for the 4th and 5th quintiles of relative humidity, respectively, vs. the first quintile of relative humidity [47].

Turbidity, or water clarity, is often used as a proxy for microbial contamination, and the EPA has established regulations limiting levels of turbidity in public drinking water [48]. EP events can increase the turbidity in surface water. However, all drinking water treatment plants in Michigan actively reduce levels of turbidity when processing raw water into finished drinking water. Studies of associations between gastrointestinal illness and turbidity, at levels as low as those measured in U.S. drinking water systems, have shown mixed results. Studies in Philadelphia, Milwaukee, Atlanta, New York City, Vancouver, and Quebec each found associations between turbidity, as measured at the treatment plant, and GI illness in subsets of age groups, subsets of seasons and/or subsets of days following the elevated turbidity, [49–51]. However, a study in Edmonton failed to find any association between turbidity and GI illness [51].

Evidence is strong for associations between GI health effects and toxins produced by cyanobacteria in harmful algal blooms (HABs) [52]. The World Health Organization has set standards for microcystin levels in public drinking water systems [52], and these levels were exceeded in the 2014 Lake Erie algal bloom in Toledo, Ohio [53]. Almost 100,000 Michigan residents use public water systems with intakes in Lake Erie. The potential health effects of HABs in these cities are of concern given that current water treatment methods do not remove all of the toxins produced by the cyanobacteria. Emergency response plans addressing a HAB, including bottled water distribution, have been put in place [53].

CSOs contaminate the receiving water with raw sewage and therefore with human pathogens. CSOs can lead to increased concentrations of pathogens in surface water [54–57]. Although treatment of the public water supply should remove these pathogens, a study in Massachusetts found associations between EP and GI illness in regions in which the public drinking water came from surface water and CSO discharges occurred [27]. We explore the implications of this finding on the present and future burden of EP-associated GI illness in our quantitative burden of disease estimate.

Flooding, which can occur during EP, may also be associated with waterborne illness. Following severe flooding in the Midwest in 2001, the EPA [58] investigated the risk of GI illness among participants of a pre-existing drinking water intervention study. The incidence rate ratio for GI symptoms during the flood vs. prior to the flood was 1.29 (95% CI: 1.06–1.58), with an increased effect among individuals with increased sensitivity to GI illness. GI symptoms were also associated with floodwater contact, particularly in children. In a case-crossover study of 129 floods in Massachusetts from 2003 to 2007, Wade et al. [59] observed an odds ratio of 1.08 (95% CI: 1.03–1.12) for ED visits for GI illness in the 0–4 day period after flooding.

People may be exposed to pathogens in contaminated surface water by ingestion, inhalation or dermal contact. These exposures may be due to 1) inadequate treatment at the water treatment plant, 2) contamination of drinking water in the treated water delivery system or 3) direct contact with contaminated surface water. Flooding, in particular, may contribute to human exposure of contaminated surface water via pathways (2) and (3) [60]. These indirect pathways between EP and illness are likely mediated by the quality of the drinking water treatment and delivery infrastructure as well as the sensitivity of the population to the microbes or their toxins.

### Multi-GCM ensemble projections

#### EH

By ZIP code, average annual EH days ranged widely, from 0 to 16 days historically and 0–46 days in the projected period (Table 2). By both ZIP code and county, the median projected average annual count of EH days was approximately 5 times higher than the historical count. The historical and projected counts of EH days were highly correlated (Spearman r = 0.99), and the six counties (St. Joseph, Wayne, Berrien, Cass, Kalamazoo, and Monroe Counties) with the highest number of EH days were the same in both the historical and projected periods (Additional file 1: Appendix 5).

**Table 2:** Minimum, median and maximum average annual number of extreme heat days, or days where the maximum temperature was 32.2–34.9 °C or ≥ 35 °C, in the historical (1971–2000) and projected (2041–2070) periods across ZIP codes and counties

	Historical			Projected		
	Minimum	Median	Maximum	Minimum	Median	Maximum
ZIP code						
32.2–34.9 °C	0.0	5.2	12.4	0.0	19.0	27.4
≥ 35 °C	0.0	0.7	3.1	0.0	7.8	18.8
Total	0	5.9	15.5	0.0	26.6	46.2
County						
32.2–34.9 °C	0.5	3.7	9.5	5.9	15.0	24.8
≥ 35 °C	0.0	0.6	2.0	1.5	6.8	14.1
Total	0.5	4.2	11.5	7.6	21.8	38.9

#### EP

In the historical period (1970–2000), there were, by definition, 7.3 EP days per year (2% of 365.25), so given an increase of 0.5–2.0 days per year statewide, EP days will increase to 7.8–9.3 days per year.

### Historical disease burden of the health outcomes

#### EH

The population age distribution changed from the historical to the projected period, with the percentage of older adults increasing from 12% to 18% statewide.

Across 83 counties, the daily baseline mortality rate for non-accidental deaths ranged from 1.8 to 5.3 deaths per 100,000 persons, with a median of 3.2 deaths per 100,000 persons in the historical period (Table 3). The median mortality rate in the projected period was slightly higher (3.3 deaths per 100,000 persons). We did not age-standardize these mortality rates, so the wide range of mortality rates partially reflects differing age distributions between counties. For renal hospitalizations, we estimated a statewide daily rate among non-whites in the warm season of 0.24 hospitalizations per 100,000 persons for both periods among individuals under 65 years of age. For older individuals, daily ED visits for non-accidental causes ranged from 34 to 56 visits per 100,000 persons in the historical period, and because of an increased percentage of older adults over time, the median in the projected period was again higher than in the historical period (53 vs. 44 visits per 100,000 persons).

**Table 3:** Minimum, median and maximum daily rate of deaths, hospitalizations, and emergency department (ED) visits across 83 Michigan counties in the historical (1971–2000) and projected (2041–2070) periods in the warm season

	Historical			Projected		
	Minimum	Median^a^	Max	Minimum	Median^a^	Maximum
Non-accidental deaths	1.8	3.2	5.3	2.0	3.3	4.8
Renal disease hospitalizations, non-whites, < 65 years old		0.57			0.57	
Renal, heat and respiratory hospitalizations, ages 65 and older	1.2	1.4	1.8	0.86	1.7	2.0
Non-accidental ED visits	34	44	56	44	53	65
Gastrointestinal illness ED visits		3.0			3.0	

aWhen only the Median value is presented, a constant rate was assumed across all counties

#### EP

For both periods, we estimated a daily ED visit rate for gastrointestinal illness in Michigan of 3 visits per 100,000 residents. Because our exposure-response association is only applicable to communities where drinking water may be exposed to CSOs, this result applies only to the 5.8 million Michigan residents served by a community public water system receiving drinking water from the Great Lakes or connecting channels or from inland rivers and lakes [53].

### Exposure-outcome associations

#### EH-mortality association

We did not see a significant association between either EH threshold (32.2–34.9, ≥35^o^ C) and mortality in the 0–4 or 5–19 age groups (Additional file 1: Appendix 4, Table A2). Among men ages 20–54, we found a risk of mortality 1.12 (1.01–1.25) times that of women during ≥35^o^ C EH days, and we found an added risk of mortality among individuals without a high school degree in this age group, as well. Among individuals 55–64, we found a 1.07 (1.00–1.14) risk of mortality during ≥35^o^ C EH vs. non-EH (Additional file 1: Appendix 4, Table A2), but we did not find significant effect modification by any of the individual or area-level characteristics tested. Among individuals ages 65 and older, we found similar risks within 10-year age groups, so these were combined as in Gronlund et al. [9]. In this age group, we found added risks among non-married individuals, and increased risk with increasing non-green space and increasing poverty at the ZIP-code level. We did not vary our AFs by marital status given that this characteristic varied little by ZIP code. Furthermore, we did not vary our AFs by non-green space given that similar land cover characteristics are used in deriving the historical and projected area-specific EH days. The increased vulnerability among residents of ZIP codes with high poverty rates was a different finding from Gronlund et al. [9], perhaps because our EH definition was more extreme than that used in Gronlund et al. [9].

For the 65 and older age group, we estimated an AF that was significantly greater than zero in 90% of the ZIP codes for EH ≥ 35 °C. Of these, the estimated AFs ranged from 0.12 to 0.69 (results not shown). Of the ZIP codes with AFs of at least 0.30, 36% were in Wayne County, MI. For EH ≥ 35 °C for men 20–49, the AF for all ZCTAs was estimated as 0.091. Likewise, for individuals ages 55–64, the statewide AF was estimated as 0.066.

#### EH-hospitalization association

Based on a statewide, all-ages RR of 1.31 for the risk of renal hospitalizations during EH days ≥32.2 °C vs. non-EH days among non-whites, we estimated an AF of 0.24 among non-whites less than 65 years of age. Among individuals 65 years and older, we estimated ZIP-code-specific AFs EH days ≥32.2 °C ranging as high as 0.63, among blacks 78 and older. Thirty-one percent of the 52 ZIP codes with AFs greater than 0.3 in any age-race group were in Wayne County, MI.

#### EH-ED visit association

Based on the RRs presented in Kingsley et al. for non-accidental ED visits, we estimated AFs for EH days 32.2–34.9 °C of 0.042 and 0.071 for the 0–18 and 65 and older age groups, respectively. For EH days ≥35 °C, we estimated slightly higher AFs of 0.052 and 0.088 for the two respective age groups. For heat-related visits in the 18–64 age group, the AFs were much higher: 0.61 and 0.69 for EH days 32.2–34.9 °C and EH days ≥35 °C, respectively.

#### EP-ED visit association

Based on an RR of 1.13 for the risk of an ED visit for a GI illness at the 99th percentile of EP, we estimated an AF of 0.12 for the residents receiving drinking water from a surface water source.

### EH- and EP-attributable health burdens

#### EH

**Fig. 2:**
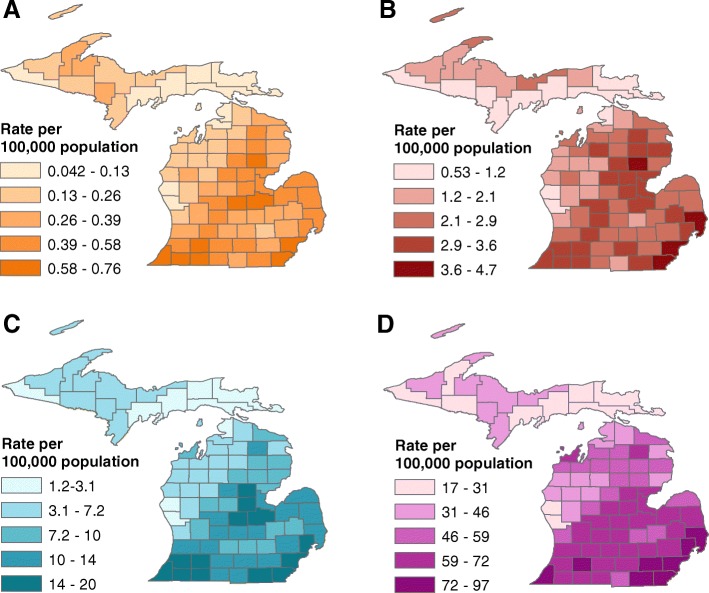
Annual heat-attributable mortality rate 1971–2000 (**a**) and 2041–2070 (**b**) and heat-attributable emergency department (ED) visit rate 1971–2000 (**c**) and 2041–2070 (**d**) by county

We estimated the rate of all non-accidental mortality associated with EH days to increase sixfold from 0.46 per 100,000 adults aged 20 years and older in the historical period (33 deaths annually statewide, Table 4) to 2.9 per 100,000 adults (240 deaths annually statewide, Table 4) in the projected period. There was significant heterogeneity between counties, with a 19-fold variation in mortality rate between counties in the historical period and a nine-fold variation between counties in the projected period (Fig. 2a-b, Additional file 1: Appendix 5).

**Table 4:** Central estimates of historical (1971–2000) and projected (2040–2070) annual counts and rates (per 100,000 persons) of disease burden and estimated cost attributable to extreme heat (EH) or extreme precipitation (EP) for the State of Michigan

Outcome	Historical Count	Historical Rate	Historical Cost^a^	Projected Count	Projected Rate	Projected Cost^a^
EH mortality	33	0.46	$42 million	240	2.9	$280 million
EH hospitalizations	28	0.28	$240,000	185	1.6	$1.6 million
EH ED visits	1200	12	$2.2 million	7800	68	$14 million
EP GI illness ED visits	170	1.7	$370,000	220	1.9	$480,000

^a^Assuming, per person: $129,000 per life-year [29] and age-specific life expectancies for persons who eventually died of circulatory or respiratory disease [34]; Michigan 2014 renal hospitalization costs of $9000 [21]; Michigan 2014 respiratory hospitalization costs of $8400 [21]; heat-related hospitalization cost of $5400 with additional costs of $1500 among ages 65–77 and $1600 among ages 78 and older {Schmeltz, 2016 #2143;{Kingsley, 2015 #1867}; non-accidental ED visit cost of $1800 and gastrointestinal infection ED visit cost of $2200, based on 2015 median costs [31]

**Fig. 3:**
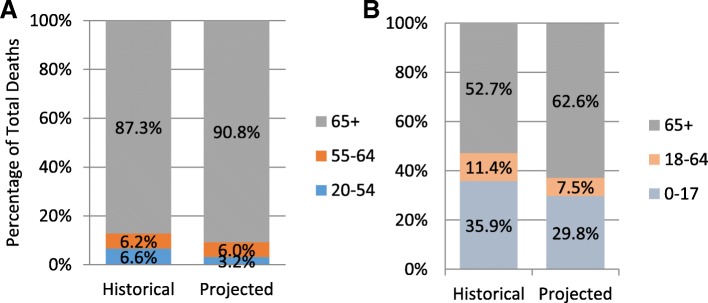
Percentage of heat-attributable deaths (**a**) and heat-attributable emergency department visit rates (**b**), by age group and time period

Mortality was highest among older adults, and the proportion of EH associated deaths that occurred in the 65 and older age group increased from the historical to the projected period (87% and 91%, respectively, Fig. 3), due to the increased percentage of the population of individuals 65 and older.

EH-associated hospitalization rates also ranged widely by county, ranging from 0.002 to 0.58 per 100,000 persons in the historical period, and 0.03 to 2.5 per 100,000 persons in the projected period. We estimated the annual statewide number of EH-attributable hospitalizations to increase from 28 in the baseline period to 185 in the projected period (Table 4).

EH-associated ED visit rates were substantially higher than EH-associated hospitalization rates, with rates of 12 per 100,000 persons (1218 visits statewide) in the historical period and 68 per 100,000 persons (7845 visits statewide) in the projected period (Table 4). Given that ED visit rates were much higher than hospitalization rates for EH, we focused the subsequent analyses and discussion of EH-associated morbidity on EH-associated ED visits. Significant heterogeneity between counties was seen, with a 16-fold variation in ED rate between counties at historical and a 6-fold variation in ED rate between counties in the projected period (Fig. 2c-d, Additional file 1: Appendix 5).

#### EP

Multiplying our estimates of the AF, EP days, GI-illness ED visit incidence rate, and population, we estimated a historical burden-of-disease rate of ED visits for GI illness attributable to EP as 170 ED visits annually, or a rate of 1.7 visits per 100,000 Michigan residents. Assuming an increase of approximately 1 day of EP in the future period, we estimated a future burden of waterborne disease attributable to EP as 220 ED visits annually, or 1.9 visits per 100,000 Michigan residents (Table 4). Considering that the number of days by which EP events will increase is projected to range spatially between 0.5 days to 2.0 days, depending on the region in Michigan, the EP-associated ED visit rate increase may range spatially from 1.0 to 3.82 visits per 100,000 Michigan residents.

#### Monetary costs

The cost of EH-associated mortality across the State of Michigan was $280 million in the projected period and $42 million in the historical period, based on a value per life-year of $129,000. The projected cost of EH-associated morbidity was dominated by EH-associated ED visits, estimated at $14 million, or $12 million higher than the historical cost (Table 4). EP costs were similar in the historical and projected periods: $390,000 and $480,000, respectively.

### Uncertainties

Table 5 summarizes the uncertainties in estimating the burden of disease due to climate.

**Table 5:** Characterization of uncertainty* by source in the estimates of the annual burden of disease attributable to extreme heat (EH) and extreme precipitation (EP) exposures in the historical (1971–2000) and projected (2040–2070) periods

Exposure and Response	Baseline health effect estimate	Population estimate	Exposure estimate	Exposure-response association	Costs
Historical					
EH-mortality	Low	Low	Low	Moderate	High
EH hospitalizations	Low	Low	Low	Moderate	Moderate
EH-ED visits	Moderate	Low	Low	Moderate	Moderate
EP-GI illness ED visits	Moderate	Low	Low	Moderate	Moderate
Projected					
EH-mortality	Moderate	Moderate	Moderate	Moderate	High
EH-renal hospitalizations	Moderate	Moderate	Moderate	Moderate	High
EH-ED visits	Moderate	Moderate	Moderate	Moderate	High
EP-GI illness ED visits	Moderate	Moderate	High	High	High

*Approximate range of the uncertainty around the central estimate: Low = ±49%, Moderate = −99% to −50% or + 50% to + 99%, High = ≤ −100% or ≥ + 200%

#### Baseline health effect estimates

For the historical estimates, our uncertainty in the baseline mortality estimates is low given that over 99% of deaths in the U.S. are thought to be registered [61]. Despite not having county-specific historical hospitalization data for our study, our estimate of uncertainty in hospitalization rates by race and age is also low, given that over 50% of the state’s black population lives in the counties for which we had detailed warm-season hospitalization rates by race and cause. Furthermore, the all-cause renal hospitalization rate in the 3 counties of 12.2% in 2014 is very close to the statewide rate of 12.5% [62]. We did not have county-specific or even statewide ED visit rates available for this study. In comparing statewide rates among the Midwest states for which data were available, we found a maximum absolute percent difference around the Midwest estimate for the heat-related ED visits of 57%, giving us moderate uncertainty in our baseline ED-visit-rate estimates (Additional file 1: Appendix 3).

Uncertainties in the projected baseline health effect estimates were moderate, based on recent trends. Age-adjusted renal hospitalization rates increased 30% over 12 years, from 100 per 100,000 persons in 2001–2003 to 130 per 100,000 persons 2012–2014. Trends in ED visit rates from 2006 to 2011 varied depending on diagnosis, with the steepest increase of 74% for septicemia [35]; but overall, ED visit rates increased 4.5% in this 6-year time period.

#### Population projections

Estimated mortality and morbidity impacts varied by population projection. The statewide EH-associated mortality rate estimated using the 2050 ICLUS A2 population estimates was 3% below that using the Woods & Poole central-case scenario while the EH-associated ED visit and hospitalization rates were 50% and 44% higher, respectively, than those using the Woods & Poole scenario. This suggests our estimates of future EH-attributable mortality are only mildly sensitive to assumptions about population growth and migration, but our morbidity estimates are moderately sensitive to population change assumptions.

#### Exposure estimates

A large source of uncertainty is that in the climate projections. The bias in number of days with maximum temperature > 32.2 °C for the A2 scenario from the six GCMs that were used to generate the Michigan-specific projections range ranged from − 50% to + 20% in Michigan. For EP, bias in wet days with > 3 in. of precipitation, the A2 scenario-specific biases range from − 90% to 100% in Michigan, depending on the GCM and the region [15]. Therefore, projections of increases could reasonably range from almost no increase in EP days to twice as many days as projected by the ensemble climate projection. Global and downscaled climate projections may contain additional biases not quantified above, especially near the Great Lakes region. The spatial resolution of GCMs does not allow for accurate representation of the lakes’ influence on local and regional climate; at best, GCMs may capture the large-scale regional effects of the lakes [63]. Furthermore, assigning EH and EP projections to ZIP codes, which are smaller than 1/8-degree, adds an additional source of uncertainty. With regards to historical EP exposure estimates, those used in deriving the precipitation exposure-response association were from single monitors rather than modeled data with high spatial resolution. Given the spatially heterogeneous nature of precipitation, this may result in bias of the effect estimates towards the null, as was demonstrated in a recent simulation study of precipitation health effects [64].

#### Exposure-response associations

Another large source of uncertainty is in our estimate of the RRs. For the EH-hospitalization estimate among individuals under 65 years of age, the 95% confidence intervals ranged from ±80% around the point estimate. Among individuals 65 and older, the 95% confidence interval for the median RR estimate (1.13) of hospitalization ranged from ±40% around the point estimate. For the EH-mortality estimates, for which we had a large Michigan-specific data set, the 95% confidence intervals around the RRs corresponded to increases of more than 100% or decreases of close to 100% in the AFs. For the EH-ED associations, the 95% confidence intervals ranged as high as 52% above the point estimate. Additionally, the cumulative effects in the days following the exposure, i.e., the lagged effects, were not estimated in the EH-ED source study, further contributing to uncertainty in the net effect of EH on ED visits.

Several recent studies have found a substantial decrease in the association between EH and mortality over time [65], suggesting strong technological and/or behavioral adaptation to EH. Nordio et al. found a decrease in the RR for mortality at 27 °C vs. 16 °C for the climate region containing Michigan, from 1.25 in the 1962–1966 time period to 1.08 in the 2000–2006 time period [66]. Likewise, Bobb et al. found that excess deaths per 1000 deaths attributable to each 5.6 °C increase in summer temperature declined from 50 per 1000 in 1987 to 11 per 1000 in 2005 in the Industrial Midwest [67]. In New York City, in a climate similar to that of Michigan’s, Petkova et al. found a decline in RR for mortality at 29 °C vs. 22 °C over 11 decades, from 1.43 in the 1900s to 1.09 in the 2000s [68]. Although all of these studies show a leveling off of this decline in recent years, changes in the RRs or attributable deaths over time of 60–80% for the mortality outcomes suggest that change over time for all of the exposure-response associations remains a source of moderate uncertainty.

External validity is another source of uncertainty in our EH-ED and EP-ED exposure-response estimates. For these estimates, we chose studies from similar climates, but population and infrastructure differences may affect the portability of RRs between two states. Michigan differs from Rhode Island in its demographic structure, with, for example, 14% vs. 8% of the population identifying as black [69]. With regards to the EP exposure-response estimate, Michigan likely differs from Massachusetts in its water delivery infrastructure, its demographic structure, and the sensitivity of its population to waterborne pathogens.

For EP, several studies of the association between precipitation and gastrointestinal illness did not find evidence of an association [28]. Jagai et al. may have been able to detect this association because they stratified their analysis by CSO exposure [27]. Additionally, in estimating the burden of disease, we applied RRs derived from a study defining EP at the 99th percentile of daily precipitation to exposure estimates of EP defined at the 98th percentile of daily precipitation, which might slightly overestimate the burden.

Communities across the State of Michigan are in the process of eliminating CSOs. However, a recent study in Massachusetts [70] found associations between sanitary sewer overflows (SSOs) and GI ED visits, suggesting that separating the sanitary and storm sewers will not entirely eliminate the pathway by which sewage can affect GI ED visits during future EP events.

#### Monetary costs

Our uncertainty in the costs associated with the historical and projected outcomes was moderate to high. For the costs associated with EH-attributable mortality, we were limited in our lack of estimates of years-of-life-lost. The time series and case-crossover study designs, on which our estimates were based, cannot estimate by how many months or years the deaths were advanced. Our approach of using the reduced life expectancies among individuals who die of cardiovascular or respiratory diseases attempts to account for this, but uncertainty in the degree of mortality displacement remains high. Uncertainty in the cost of a life-year is also high, with a reported range of $65,000 to $490,000 [29]. For the morbidities, uncertainties in the estimated costs were moderate. The reported ranges were less than ±50% around the point estimates for hospitalization costs [30], but we are uncertain as to how well the estimates reflected the cost of an EH-associated visit that was not necessarily coded as heat-related. Specifically, we used heat-related hospitalization costs to estimate EH-associated renal and respiratory hospitalizations, and we used non-accidental ED visit costs to estimate ED visits that were presumably triggered by EH.

We have additional uncertainty in projecting the costs given trends in medical costs over time. For example, in 2011 dollars, the average cost of an ED visit for individuals age 65 and older increased 40% over just 10 years from $630 in 2001 to $880 in 2011 [71]. Similarly, daily inpatient costs increased 30% from $2400 to $3200.

## Discussion

The health burden due to EH and EP may significantly rise for all Michigan counties by midcentury, with the greatest mortality and ED-burden in the southeast. To provide a sense of the public health significance of our projections, the projected combined EH- and EP-attributable ED burden in Southeast Michigan, as high as 0.97/1000 persons, is not as high as the 2012–2013 national motor-vehicle accident ED visit rate of 10/1000 persons [72], but it is comparable to the 2014 national ED-visit rate for miscarriages of 1.2/1000 persons [73]. Adaptation measures against extreme temperatures are needed to protect health, such as targeted public health monitoring, expanded access to air conditioning, and reductions in the urban heat island effect. Health protection of older adults should be prioritized in public health planning given the large burden among those aged 65 and older.

These estimates represent the total non-accidental effects of EH as identified in the Michigan-specific causal pathway, including effects which may be mediated by ozone. It is not likely that ozone strongly mediates the EH-mortality or EH-hospitalization effects given that its inclusion in models of the association between EH and mortality and hospitalizations in Michigan did not affect the estimates of the EH effects by more than 10% [9, 18]. We did not have projected increases in ozone concentrations and could not estimate its projected direct effect, independent of EH. However, a projection of mortality in 2041–2050 due to ozone in 19 communities in the Southeastern U.S. found a small, 0.43 ppb increase in average ozone concentration due to climate change as compared to concentrations in 2000 and a concomitant 0.01% increase in the mortality rate attributable to climate-change related increases in ozone [74]. A study of the New York City metropolitan region projected a 7.3% increase in ozone-related asthma emergency department visits among children by the 2020s as compared to 1990s [75], suggesting modest independent effects of ozone.

In addition to potential increases in ozone, some of the historical and projected ED visits which we attributed to EH may be among individuals experiencing asthma exacerbations. Several studies have found associations between temperature and asthma symptoms or ED visits. Winquist et al. found a 6% increase in the risk of ED visits for asthma among children in Atlanta at the 75th vs. 25th percentile of daily maximum temperature [76]. In Australia, Li et al. found increased reports of wheeze/chest tightness and cough/phlegm with increasing temperatures in a cohort of 270 children with asthma [77], and pediatric ED visits for chronic lower respiratory diseases increased in association with high temperatures in a separate study in Brisbane, Australia [78]. Hospitalizations were increased for asthma and other respiratory conditions in New York City [79]. An evaluation of pediatric records from a Detroit hospital found an additional 1.8 asthma ED visits for a 10 °F increase in temperature [80]. We did not quantify the specific morbidity burden of asthma attributable to EH, but given the high prevalence of asthma in the State of Michigan [81], EH-associated asthma morbidity may be of particular concern in Michigan with climate change.

Although reviews on preterm birth and heat published prior to this project’s scoping phase suggested that the association was unclear [82, 83], several studies have been published more recently indicating an association between birth outcomes and heat [84–94]. Future burden of disease assessments in Michigan should consider birth outcomes, particularly in light of the high baseline preterm birth rate in Detroit [95]. Similarly, we did not identify injuries, including occupational injury not listed as heat-related, or self-inflicted injuries as EH-associated concerns in the scoping phase. Recent research indicates significant associations of non-heat-related injuries with EH [96, 97], and future Michigan climate burden of disease assessments should consider EH-associated injuries.

Climate change may have significant impacts on public health beyond EH and EP impacts beyond those quantified in this paper. Notably, pollen levels are expected to increase in North America [98]. Temperature and precipitation, as well as carbon dioxide concentrations, affect the levels of several types of tree and grass pollen, and this may lead to increases in allergic respiratory morbidity [99]. Furthermore, the pollen season length is increasing with climate change, although research on pollen trends is lacking due to a lack of consistent pollen monitoring over a long timescale [98]. Future research should address the impacts of higher pollen levels on asthma and allergy incidence and exacerbation.

We have only calculated the EP-attributable burden of GI illness related to ED visits. Most people experiencing GI illness do not seek treatment in EDs or medical care of any kind. Using a variety of surveillance systems, Mead et al. estimated that the average person experiences 1.05 episodes of GI illness annually characterized by diarrhea, vomiting or both [100]. By this estimate, GI-related ED visits *underestimate* the total burden of GI-related illness by a factor of 100, and our estimate of the burden of GI illness attributable to EP is severely underestimated by our EP-attributable burden of GI-related ED visits. Furthermore, our quantitative EP estimate may not have accounted for the effects of harmful algal blooms, which may affect Michigan and Massachusetts drinking water delivery systems differently.

We did not project increases in legionellosis with EP given the inconsistency in the literature in associations between legionellosis and precipitation. However, some of the studies suggested a greater importance of temperature and humidity than EP in legionellosis incidence, and confidence that temperature will increase with climate change is high.

Several limitations to our estimates of the burden of future EH and EP exist, largely stemming from uncertainty surrounding various model inputs. The uncertainty analysis indicated that our mortality projections are only mildly sensitive to different population assumptions. Other inputs, including the exposure data and exposure-response function, carry greater uncertainty. A key limitation in our approach is the use of historical relationships for the estimation of future health effects without accounting for long-term adaptation to EH or EP. Physiological and behavioral adaptations have the potential to reduce the impact of extreme weather. Our results may therefore be regarded as a direr scenario where effective public health measures protecting individuals from extreme weather events have not been adopted.

Additionally, our analysis does not fully account for uncertainty in climate models or future climate conditions. Our projections of EH days were derived using the high emissions A2 scenario, although the lower emissions scenarios are becoming statistically improbable given recent historic emissions trends [101]. Incorporating exposure projections derived from alternate, low emissions scenarios would likely result in lower mortality estimates. On the other hand, evidence from other studies suggests that mortality and morbidity increase at less extreme temperatures in temperate climates such as Michigan [8, 25]. This would imply that our results underestimate the total number of deaths and ED visits associated with this climate scenario.

## Conclusions

Under a dire climate change scenario, we estimated a Michigan-wide increase in EH-associated mortality from 0.46/100,000 persons historically to 2.9/100,000 in the projected period for $280 million in costs. We estimated a more substantial increase in EH-associated ED visits from 12/100,000 persons to 68/100,000 persons, for 7800 EH-attributable emergency department visits and $14 million in costs. EP-associated ED visits increased minimally from 1.7 to 1.9/100,000 persons, although this quantitative estimate did not include self-treated gastroenteritis or sufficiently represent the range of health problems from harmful algal blooms, flooding, and legionellosis. With the use of a high-emissions climate scenario and the exclusion of adaptation in the model we may over-estimate the future burden due to extreme temperatures in Michigan, but the exclusion of health effects from moderate heat, pollen, and precipitation-associated respiratory effects as well as any non-emergent health effects may result in substantial underestimation of both the present and future burden of climate in Michigan. Further research should investigate the association between moderate heat, precipitation, pollen, and health to gain a more complete picture of Michigan’s climate-related disease burden. Our finding of a notable burden of mortality and morbidity attributable to EH, without assumptions about adaptation, stresses the importance of actions to protect health against the adverse health effects of EH. Additionally, although the uncertainty in the GI-illness projections is high, the health risks of poor public drinking water quality that can result from harmful algal blooms and storm-related emergencies are clear. In these emergencies, access to clean water for drinking and bathing can become challenges, and preparations for increases in EP events should focus on distribution of clean, potable water in emergencies to affected residents to avoid waterborne illnesses.

## Additional file

Additional file 1: **Appendix 1.** Additional details on methods [104]. **Appendix 2.** Deriving ZCTA-level estimates for EH days. **Appendix 3.** Derivation of ED visit rates. **Appendix 4**. Heat-mortality epidemiologic results. **Appendix 5.** County-specific inputs and results [105]. **Appendix 6.** Loading data into BenMAP. (DOCX 75 kb)

## Abbreviations

AF: Attributable fraction
AFD: Attributable fraction day
BenMAP: Environmental Benefits Mapping and Analysis Program
BOD: Burden of disease
ED: Emergency department
EH: Extreme heat
EP: Extreme precipitation
EPA: U.S. Environmental Protection Agency
GCM: Global climate model
GI: Gastrointestinal
HAB: Harmful algal bloom
HCUP: Healthcare Cost and Utilization Project
ICD: International Classification of Diseases
ICLUS: Integrated Climate and Land-Use Scenarios
QALY: Quality-adjusted life year
RR: Risk ratio
ZCTA: ZIP code tabulation area
