# Supplementary material for: Assessing the magnitude and uncertainties of the burden of selected diseases attributable to extreme heat and extreme precipitation under a climate change scenario in Michigan for the period 2041–2070
Source: Environ Health. 2019 Apr 27;18:40. doi: 10.1186/s12940-019-0483-5 (PMC6487044; doi:10.1186/s12940-019-0483-5)
Supplement: Supplementary file 1 — Appendix 1. Additional details on methods [104]. Appendix 2. Deriving ZCTA-level estimates for EH days. Appendix 3. Derivation of ED visit rates. Appendix 4. Heat-mortality epidemiologic results. Appendix 5. County-specific inputs and results [105]. Appendix 6. Loading data into BenMAP. (DOCX 75 kb) [file 12940_2019_483_MOESM1_ESM.docx]

# Additional file

## Appendix 1. Additional Details on Methods

## 3. Historical disease burden of the health outcomes

### Mortality rates

BenMAP used census projections of mortality rates to estimate the mortality rate in 5-year increments to the year 2050 [17]. In order to capture mortality rates within the ranges of historical and projected climate information, all non-accidental mortality rates (International Classification of Diseases (ICD)-10 A00-R99) for the year 2000 were used for the historical and projected analyses.

### *Hospitalization rates*

Annual warm season (May-September) renal hospitalization rates were estimated by dividing warm-season renal hospitalization counts from a 3-county study of EH associated hospitalizations in Michigan [18] by U.S. Census population estimates [19] for rates of 8.78 per 10,000 persons of non-white race among individuals under 65 years of age, or 0.57 per 100,000 persons daily (Table 3). Hospitalizations for renal, respiratory, or heat-related causes among individuals 65 and older were estimated by dividing the age-race group specific warm-season hospitalization counts from the 7 counties in Michigan included in a nationwide study of EH-associated hospitalizations [20] by U.S. Census population estimates [19] (Table 3).

### ED rates

Additional details and a validation of the derived data are provided in Appendix 3.

### Population counts

BenMAP’s population projections utilize population data from the 2000 U.S. Census [16], which were projected to 2050 using growth factors based on the Woods & Poole economic forecasting model [17]. In order to temporally match mortality incidence and climate projection data, population projections for the year 2050 were obtained from the EPA’s Integrated Climate and Land-Use Scenarios (ICLUS) project for the A2 scenario and used in place of the pre-loaded 2050 population projections [16,103].

## 4. Historical exposure-outcome associations

### EH-mortality association

To estimate the EH-mortality association, we performed a new epidemiologic analysis based on a recent Michigan-specific analysis by Gronlund et al [9]. As in Gronlund et al., National Land Cover Dataset land cover classifications for 1992, 2001 and 2006 were obtained. The percent of non-green space was estimated for each ZIP code as 100 – (percent of 30 x 30 m cells that we categorized as green space). The following Census/American Community Survey ZIP-code-level estimates were obtained for the years 1990, 2000 and 2010: total population count, percent black, percent of individuals 65 years and older and living alone and percent below the poverty level. The ZIP-code-level land cover and demographic characteristics were linearly interpolated such that a value was available for each year between 1990 and 2007. These characteristics were merged, by ZIP code and year, with Michigan death records from 1990-2007, which contained the date of death, age, race (further categorized as black vs. non-black), educational attainment (further categorized as high school vs. no high school), sex and marital status of the decedents.

Following the modeling in Gronlund et al. [9], within each age category of 0-4, 5-19, 20-49, 50-64, 65-74, 75-84, 85 years or older and 65 years or older, we estimated the cumulative association between EH, for 0-3 days following the EH day, and mortality in May-September, in 8 cities, aggregated from 10 counties, in Michigan. We used a case-crossover model using conditional logistic regression with interaction terms between EH (the 4-day sum of daily EH indicators) and black race, no high school diploma, non-married, aged 75 or older, male sex, percent non-green space, percent of residents 65 and older living alone, percent of residents below the poverty level, and city. The inclusion of multiple interaction terms in a single model allowed for control for confounding among these potential effect modifiers. Each potential modifier was mean-centered and standardized for an interquartile-range increase so that the coefficient for the main effect of EH represented the effect among an average individual in the dataset. This modeling differed from that in Gronlund et al. 2014 in three ways. Firstly, EH was defined as a step function of daily maximum temperature, with steps at 32.2 °C and 35 °C (indicator variables for 32.2-34.9 °C and 35 °C or higher). Secondly, the outcome was all-natural-cause mortality (ICD-9 codes < 800, roughly equivalent to ICD-10 A00-R99) and heat-related codes E900.0 and 992 rather than mortality for which the principal cause-of-death was cardiovascular or respiratory disease. Finally, all ages were studied as opposed to restricting the analysis to individuals 65 years and older.

Attributable fractions (AFs) for the cumulative effects of one day of EH were calculated for each county as follows. For each person *i* in ZIP code *z,* age group *a* and EH threshold *t*, a mortality risk ratio (*RR*) for EH vs. non-EH was estimated as:

${RR}_{izat}= e^{\beta_{1}\times EH+\beta_{2}\times EH\times{MALE}_{i}+\beta_{3}\times EH\times{POV}_{z}+\beta_{4}\times{EH\times NOHS}_{i}}$ Equation 1

where *β_1_*, *β_2,_* *β_3_* and *β_4_* were the coefficients from the case-crossover model for EH, male sex, poverty and no high school, respectively, *EH* was the four-day sum of dummy variables indicating whether maximum temperature was above the EH threshold and was set equal to 4, *MALE* was set to equal 1 for males and 0 for females, *POV_z_* was the percentage of residents below the poverty level in 2000 in ZIP code *z* and *NOHS* was 1 for individuals without a high-school education in 2000 and 0 otherwise. When *β_2_, β_3_* or *β_4_* were not statistically significant, that particular coefficient was set to zero. Standard errors were estimated and non-significant RRs, i.e., those with z-scores less than 1.96, were set to 1. AFs for each ZIP code *z,* age group *a* and extreme-heat threshold *t* were estimated as:

${AF}_{zat}=1-\sum\frac{{pNoHS}_{z}}{{RR}_{zat}}$ Equation 2[104]

where *pNoHS_z_* was the proportion of cases in ZIP code z without a high school education as derived from the high-school attainment variable in the mortality records. These were interpreted as the fraction of deaths in age group *a* in ZIP code *z* attributable to one day of EH for threshold *t*.

### EH-hospitalization association

In three Michigan counties, Ogbomo et al. [18] found a significant association with EH only for hospitalizations for renal diseases (ICD9-CM 580-589) and only among non-whites. From Ogbomo et al., we estimated the RRs for individuals under 65 years of age on EH days vs. non-EH days at EH thresholds of 32.2^o^ C, which corresponded to the 95th percentile of daily maximum temperature in the study. These were estimated as the 97th percentile threshold estimate among non-whites from Ogbomo et al., 1.37 (95% confidence interval: 1.13, 1.66), divided by the ratio of the 97th to 95th percentile threshold estimates among all races (1.19 / 1.14), for an estimate of 1.31 and an AF of 0.24. For individuals 65 years of age and older, we used the air conditioning prevalence region 2 (which included Michigan) estimates from the U.S. study of effect modification of renal, respiratory and heat-related hospitalizations by Gronlund et al. [20] Specifically, we used the effect estimates (betas) of EH at the 97^th^ percentile of maximum daily temperature, which was within 1 degree of 32.2^o^ C for the Michigan counties included in the study, vs. the 75^th^ percentile of temperature, as well as the modifying effects of black race (*BLACK*), age greater than 77 years (*AGE78UP*), and the proportion of residents of the ZCTA without a high school education (*NOHS*) and living in homes built before 1940 (*BUILT39*). Similarly to the mortality estimates above, for each time period we modeled the 6-day cumulative effects of one day of EH vs. non-EH (mean of maximum temperature on lag days 0-1 > 97^th^ percentile (EH01) and mean of maximum temperature on lag days 2-5 > 97^th^ percentile EH25)) as:

${RR}_{z}= e^{\begin{aligned} \beta_{1}\times EH01+\beta_{2}\times EH01\times BLACK+\beta_{3}\times EH01\times AGE78UP+\beta_{4}\times{EH01\times NOHS}_{z}+\beta_{5}\times EH01\times BUILT39+ \\ \beta_{6}\times EH25+\beta_{7}\times EH25\times BLACK+\beta_{8}\times EH25\times AGE78UP+\beta_{9}\times{EH25\times NOHS}_{z}+\beta_{10}\times EH25\times BUILT39 \end{aligned}}$`Equation 3

for each combination of *AGE78UP* and *BLACK* in each ZCTA *z*.

### EH-ED visit association

Exposure-response functions were derived from a study examining the effects of heat on morbidity and mortality in Rhode Island [25], a state with a climate similar to that of Michigan’s [26]. By averaging across temperature categories, RRs for the rate of all-natural-cause ED visits in April-October 2005-2012 for 32.2 °C relative to 21.1 °C were estimated as 1.044 in the 0-18 age group and 1.077 in the 65 and older age group. For 35 °C relative to 21.1 °C, RRs were estimated as 1.055 in the 0-18 age group and 1.097 in the 65 and older age group. For the 18-64 age group for heat-related ED visits and for 32.2 °C and 35 °C relative to 21.1 °C, we estimated RRs of 2.56 and 3.24 for AFs of 0.61 and 0.69, respectively.

### EP-ED visit association

A recent review of 24 studies of the association between EP and waterborne infections [28] suggested that the heterogeneity observed among the studies’ results may be attributed to the type of water supply, water source, or water treatment, among other variables. The RR estimated in Massachusetts [27] for the cumulative effect of one day of EP (defined as the 99th percentile of daily precipitation) and ED visits for GI illness in regions where the drinking water was exposed to CSOs was 1.13 (95% Confidence Interval: 1.00-1.28). However, there were no statistically significant associations between EP and ED visits for GI illness at less extreme thresholds of EP (90th or 95th percentiles of daily precipitation) or in regions where only the recreational water was exposed to CSOs or there were no CSOs. In the Massachusetts study there was no statistically significant evidence of effect modification by age category, although RRs were positive in all age groups and highest (1.32, 95% CI: 0.92-1.88) among individuals aged 65 and older [27].

## Appendix 2: Deriving ZIP Code Tabulation Area (ZCTA)-level estimates for extreme heat (EH) days

Data were aggregated to 2000 Census ZIP Code Tabulation Areas (ZCTAs) to match the spatial resolution of the health data. ZCTAs are polygons which correspond to U.S. postal codes, or ZIP codes. These steps were performed separately for the historical (1971-2000) and projected (2041-2070) datasets. The data were aggregated using the zonal statistics function in ArcGIS v10.1

1. We downloaded a TIGER/Line shapefile from the Census website for the 2000 Census ZCTAs.
   - <https://www.census.gov/geo/maps-data/data/tiger-line.html>
2. We imported climate projection data into ArcGIS. For this analysis, data from the National Climate Assessment were provided by GLISA at a resolution of 1/8° in the .nc format, and were converted to a raster for use in ArcGIS.
   - ArcToolbox > Multidimension Tools > Make NetCDF Raster Layer
3. We copied the raster layer in order to assign 'background value' and 'no data value' to -999. This prevented missing value raster cells (coded as -999) from contributing to zonal statistics calculations.
   - ArcToolbox > Data Management Tools > Raster > Raster Dataset > Copy Raster
4. We used the zonal statistics tool to find a mean value of EH days for each ZCTA., utilizing the copied layer from step 3 and selecting “Ignore no data”. This created a visual representation of the mean number of EH days.
   - ArcToolbox > Spatial Analyst Tools > Zonal > Zonal Statistics
5. We converted the zonal statistics layer to a table using the copied layer from step 3.
   - ArcToolbox > Spatial Analyst Tools > Zonal > Zonal Statistics as Table
6. We exported the table created in step 5 to Excel so that the data could be imported into SAS.
   - ArcToolbox > Conversion Tools > Excel > Table To Excel

## Appendix 3: Derivation of Emergency Department (ED) Visit Rates

### Methods

For consistency with existing estimates in BenMAP, we replicated the procedure used by BenMAP to estimate state-level ED visit rates [17] for diagnoses specific to those used in Kingsley et al. [25] in modeling the EH-ED exposure response association.

1. We obtained the endpoint-specific ED visit counts in the Midwest region from HCUPnet/NEDS, or the HCUP National Emergency Department Sample, which weights the samples by hospital region, trauma center designation, urban/rural location, teaching status, and ownership. We refer to this count as TOTAL.
   1. States in the Midwest region include Illinois, Indiana, Iowa, Kansas, Minnesota, Missouri, Michigan, Nebraska, North Dakota, Ohio, South Dakota, and Wisconsin, although only Illinois, Iowa, Minnesota, and Nebraska contribute data.
2. For those states in the Midwest region that do have discharge-level or state-level data in the State Emergency Department Database, we summed the ED visits by endpoint (we refer to this count as SUB).
   1. States in the Midwest region that do have discharge-level or state-level data in the HCUPnet central portal include Illinois, Iowa, Minnesota, and Nebraska.
      1. In the HCUPnet query for ‘State Statistics on All ED Visits’, select ‘All ED visits’ and ‘First-listed diagnosis’. Selecting ‘All ED visits’ includes both discharges from the ED and ED visits with admission to the same hospital.
      2. The data year varies across states from 2007 (Iowa, Minnesota, Nebraska) to 2009 (Illinois); we assumed that ER visit rates are reasonably constant across these two years and consider them as 2007 rates. The year 2007 was chosen to match rates already in BenMAP.
      3. ICD-9 codes were selected to match those in Kingsley et al. [25]:
         1. Heat: E900, 992, 276.51
         2. All-cause: <800, E900, 992
3. We then estimated the ED visit count for states without discharge or state data for each endpoint as TOTAL - SUB. Note that while this count is endpoint- and region-specific, it is not age-specific. We obtained the distribution of ED visit counts across age groups based on the HCUP National Statistics and assumed the same distribution for the Midwest Region. We then applied this distribution to the estimated ED visit counts (i.e., TOTAL - SUB) to obtain endpoint- and age-specific counts.
   1. States in the Midwest region without discharge-level or state-level data in the HCUPnet central portal include Indiana, Kansas, Missouri, Michigan, North Dakota, Ohio, South Dakota, and Wisconsin. We refer to these states as “no-data” states.
4. We calculated age-specific and endpoint-specific ED visit rates for by dividing TOTAL-SUB by the 2007 Census age-specific populations for the “no-data” states. The resulting regional rate was applied to all Michigan counties.

### Variability in state-specific data in the Midwest region

We did not have Michigan ED data with which we could validate our approach. Therefore, to estimate the variability in ED visit rates between states, we estimated the percent difference in ED visit rates for non-accidental, heat-related and gastrointestinal (GI) causes in each available Midwest state between 1) the state’s estimate in the State Emergency Department Database and 2) the Midwest region estimate in the National Emergency Department Sample. The maximum absolute percent difference for the all-non-accidental causes was less than 50% of the Midwest estimate for ages 0-17 and ages 65 years and older. The maximum absolute percent difference around the Midwest estimate for the heat-related ED visits was higher, with Illinois’s heat-related ED visit rate at 57% less than the Midwest estimate.

Table A1. Non-accidental, heat-related and gastrointestinal (GI) emergency department (ED) visit counts, rates for four Midwestern states for which data were available and regional Midwestern estimates based on a national sample.

|  | ED visit counts | | | | ED visit rates (per 1,000 persons)^1^ | | | |
| --- | --- | --- | --- | --- | --- | --- | --- | --- |
|  | ages 0-17 | ages 18-64 (heat-related) | ages 65+ | All ages GI | ages 0-17 | ages 18-64 (heat-related) | ages 65+ | All ages GI |
| Illinois | 863,830 | 1,742 | 336,886 | 130,635 | 70 | 0.13 | 26 | 10 |
| Iowa | 158,603 | 415 | 115,090 | 33,302 | 57 | 0.15 | 32 | 11 |
| Minnesota | 239,768 | 819 | 135,773 | 43,560 | 48 | 0.16 | 26 | 8 |
| Nebraska | 81,674 | 436 | 49,131 | 14,669 | 44 | 0.25 | 26 | 8 |
| Midwest^2^ | 4,072,359 | 20,464 | 2,065,981 | 801,889 | 64 | 0.31 | 30 | 12 |
| Uncertainty range^3^ |  |  |  |  | -32%, 8% | -57%, -19% | -14%, 8% | -32%, -7% |

^1^Standardized to a standard 2000 U.S. Population

^2^The age-group specific results are calculated as the all-ages total multiplied by the percent of ED visit counts in each age group in the national average.

^3^ Range of percent difference between state estimates and Midwest estimate.

## Appendix 4. Heat-mortality epidemiologic results.

Table A2. Overall and added risk (95% confidence intervals) of all-natural cause mortality during extreme heat (daily maximum temperature 32.2-34.9 °C or ≥ 35 °C) for the presence vs. absence or interquartile-range (IQR) increase in the characteristic, by age group, in 8 Michigan cities, May-September, 1990-2007.

|  |  | Age Group | | | |
| --- | --- | --- | --- | --- | --- |
| Characteristic | Extreme heat (°C) | 0-4 Years | 20-54 Years | 55-64 Years | 65 + Years |
| Overall | 32.2-34.9 | 0.98 (0.93, 1.03) | 1.01 (0.99, 1.03) | 1.00 (0.98, 1.02) | **1.01 (1.01, 1.02)** |
|  | ≥ 35 | 0.86 (0.69, 1.06) | 1.05 (0.99, 1.12) | **1.07 (1.00, 1.14)** | **1.06 (1.04, 1.08)** |
| Black | 32.2-34.9 | 0.97 (0.87, 1.08) | 1.02 (0.98, 1.06) | 1.01 (0.96, 1.05) | 1.02 (1.00, 1.04) |
|  | ≥ 35 | 0.87 (0.60, 1.27) | 0.99 (0.86, 1.14) | 0.99 (0.84, 1.16) | 0.98 (0.91, 1.05) |
| No high school | 32.2-34.9 |  | **1.04 (1.00, 1.08)** | 0.98 (0.94, 1.02) | 1.00 (0.98, 1.01) |
|  | ≥ 35 |  | 1.04 (0.92, 1.17) | 0.91 (0.80, 1.03) | 1.01 (0.97, 1.05) |
| Non-married | 32.2-34.9 |  | 0.99 (0.95, 1.02) | 1.01 (0.98, 1.04) | 1.00 (0.99, 1.02) |
|  | ≥ 35 |  | 1.07 (0.95, 1.19) | 1.03 (0.93, 1.16) | **1.08 (1.03, 1.15)** |
| Aged 75+ | 32.2-34.9 |  |  |  | 1.01 (0.99, 1.02) |
|  | ≥ 35 |  |  |  | 0.98 (0.93, 1.03) |
| Male | 32.2-34.9 | 1.00 (0.98, 1.01) | 1.01 (0.98, 1.05) | 0.98 (0.95, 1.01) | 1.00 (0.98, 1.01) |
|  | ≥ 35 | 1.04(0.99, 1.09) | **1.12 (1.01, 1.25)** | 0.94 (0.85, 1.05) | 1.04 (0.99, 1.09) |
| % Non-green space (IQR = 51.9%) | 32.2-34.9 | 0.99 (0.88, 1.11) | 1.02 (0.98, 1.06) | 0.99 (0.95, 1.03) | 1.01 (0.99, 1.02) |
|  | ≥ 35 | 1.01 (0.70, 1.44) | 0.91 (0.79, 1.04) | 1.02 (0.89, 1.17) | **1.08 (1.03, 1.15)** |
| % Aged 65+ and alone (IQR = 3%) | 32.2-34.9 | 1.00 (0.95, 1.06) | 0.99 (0.97, 1.01) | 1.01 (0.99, 1.03) | 1.00 (0.99, 1.00) |
|  | ≥ 35 | 0.89 (0.74, 1.08) | 1.01 (0.95, 1.08) | 0.94 (0.88, 1.01) | 0.98 (0.95, 1.00) |
| % Below poverty level (IQR = 12.8%) | 32.2-34.9 | 0.99 (0.94, 1.05) | 0.99 (0.96, 1.01) | 1.00 (0.98, 1.03) | 1.01 (1.00, 1.02) |
|  | ≥ 35 | 1.20 (0.99, 1.45) | 1.02 (0.95, 1.11) | 1.01 (0.93, 1.09) | **1.03 (1.00, 1.07)** |

p < 0.05 for bolded values.

For 5-19 years of age, the RR for the 32.2-34.9 °C extreme heat range was 1.00 (0.90, 1.11) and there were an insufficient number of decedents on extreme heat days 35 °C or higher to permit analysis.

## Appendix 5: County-Specific Inputs and Results

Table A3. Extreme heat (EH) days and attributable fraction days (AFDs) in the historical period (1971-2000) for mortality, emergency department (ED) visits, and renal, respiratory and heat-related hospital admissions (HA).

|  | EH days | |  | Mortality AFDs | | | ED visit AFDs | | | HA AFDs | | | | |
| --- | --- | --- | --- | --- | --- | --- | --- | --- | --- | --- | --- | --- | --- | --- |
| County | 32.2-34.9 | 32.2-34.9 | men ages 20-54 | women ages 20-54 | ages 55-64 | ages 65+ | ages 0-17 | ages 18-64 (heat-related) | ages 65+ | non-white, ages 0-64 | ages 65-77 nonblack | ages 65-77 black | ages 78+ nonblack | ages 78+ black |
| Alcona | 2.6 | 0.4 | 0.18 | 0.04 | 0.1 | 0.2 | 0.13 | 1.87 | 0.22 | 1.14 | 0.06 | 0.23 | 0.28 | 0.48 |
| Alger | 1.8 | 0.5 | 0.16 | 0.01 | 0.11 | 0.17 | 0.10 | 1.43 | 0.17 | 0.77 | 0.01 | 0.21 | 0.17 | 0.4 |
| Allegan | 5.7 | 0.6 | 0.25 | 0.04 | 0.16 | 0.35 | 0.27 | 3.88 | 0.46 | 2.36 | 0.09 | 0.65 | 0.58 | 1.01 |
| Alpena | 2.5 | 0.5 | 0.18 | 0.01 | 0.13 | 0.21 | 0.13 | 1.88 | 0.22 | 1.18 | 0.03 | 0.32 | 0.3 | 0.57 |
| Antrim | 3.0 | 0.2 | 0.09 | 0.02 | 0.05 | 0.17 | 0.14 | 1.97 | 0.23 | 1.07 | 0.02 | 0.2 | 0.21 | 0.45 |
| Arenac | 3.9 | 0.7 | 0.32 | 0.07 | 0.18 | 0.33 | 0.21 | 2.98 | 0.35 | 1.87 | 0.09 | 0.73 | 0.49 | 1.12 |
| Baraga | 2.4 | 0.5 | 0.19 | 0.02 | 0.13 | 0.21 | 0.13 | 1.89 | 0.23 | 1.33 | 0.12 | 0.47 | 0.41 | 0.75 |
| Barry | 6.5 | 1.0 | 0.37 | 0.07 | 0.23 | 0.44 | 0.32 | 4.59 | 0.54 | 2.84 | 0.1 | 0.96 | 0.69 | 1.59 |
| Bay | 6.1 | 0.9 | 0.33 | 0.03 | 0.22 | 0.44 | 0.31 | 4.43 | 0.52 | 2.61 | 0.14 | 0.71 | 0.61 | 1.35 |
| Benzie | 2.7 | 0.3 | 0.09 | 0 | 0.06 | 0.16 | 0.13 | 1.90 | 0.22 | 0.81 | 0 | 0.17 | 0.11 | 0.45 |
| Berrien | 8.0 | 1.3 | 0.54 | 0.14 | 0.3 | 0.61 | 0.40 | 5.79 | 0.69 | 3.81 | 0.19 | 1.47 | 0.89 | 2.14 |
| Branch | 5.8 | 0.8 | 0.32 | 0.08 | 0.18 | 0.39 | 0.28 | 4.02 | 0.48 | 3.08 | 0.38 | 0.87 | 0.94 | 1.38 |
| Calhoun | 6.2 | 0.9 | 0.36 | 0.07 | 0.22 | 0.44 | 0.30 | 4.35 | 0.52 | 2.90 | 0.16 | 0.91 | 0.73 | 1.48 |
| Cass | 8.1 | 1.3 | 0.55 | 0.14 | 0.31 | 0.61 | 0.40 | 5.76 | 0.68 | 3.99 | 0.28 | 1.18 | 1.09 | 1.93 |
| Charlevoix | 2.5 | 0.2 | 0.07 | 0.01 | 0.04 | 0.14 | 0.11 | 1.63 | 0.19 | 0.94 | 0.01 | 0.21 | 0.2 | 0.39 |
| Cheboygan | 2.3 | 0.2 | 0.08 | 0.01 | 0.05 | 0.14 | 0.10 | 1.49 | 0.18 | 0.92 | 0.02 | 0.32 | 0.22 | 0.57 |
| Chippewa | 0.6 | 0.1 | 0.04 | 0.01 | 0.02 | 0.05 | 0.03 | 0.45 | 0.05 | 0.28 | 0.02 | 0.07 | 0.07 | 0.15 |
| Clare | 4.5 | 0.8 | 0.36 | 0.1 | 0.2 | 0.39 | 0.23 | 3.34 | 0.40 | 2.00 | 0.02 | 0.51 | 0.49 | 0.94 |
| Clinton | 5.7 | 0.8 | 0.27 | 0.01 | 0.2 | 0.42 | 0.28 | 4.02 | 0.48 | 2.20 | 0.12 | 0.55 | 0.45 | 1.01 |
| Crawford | 2.8 | 0.6 | 0.22 | 0.04 | 0.13 | 0.24 | 0.14 | 2.06 | 0.24 | 1.13 | 0 | 0.28 | 0.23 | 0.58 |
| Delta | 0.6 | 0.0 | 0.01 | 0 | 0.01 | 0.03 | 0.03 | 0.44 | 0.05 | 0.28 | 0.01 | 0.07 | 0.08 | 0.11 |
| Dickinson | 2.7 | 0.5 | 0.15 | 0 | 0.11 | 0.21 | 0.14 | 1.94 | 0.23 | 1.25 | 0.05 | 0.36 | 0.33 | 0.61 |
| Eaton | 5.2 | 0.7 | 0.25 | 0.02 | 0.18 | 0.35 | 0.26 | 3.67 | 0.43 | 2.06 | 0.11 | 0.34 | 0.43 | 0.83 |
| Emmet | 1.4 | 0.1 | 0.03 | 0 | 0.02 | 0.08 | 0.07 | 0.95 | 0.11 | 0.50 | 0 | 0.07 | 0.1 | 0.15 |
| Genesee | 4.7 | 0.8 | 0.28 | 0.04 | 0.18 | 0.36 | 0.24 | 3.40 | 0.40 | 1.91 | 0.04 | 0.78 | 0.31 | 1.16 |
| Gladwin | 5.3 | 1.0 | 0.4 | 0.08 | 0.24 | 0.45 | 0.28 | 3.99 | 0.47 | 2.36 | 0.04 | 0.55 | 0.57 | 1.11 |
| Gogebic | 1.1 | 0.1 | 0.03 | 0 | 0.02 | 0.07 | 0.05 | 0.74 | 0.09 | 0.58 | 0.08 | 0.14 | 0.18 | 0.24 |
| Grand Traverse | 3.4 | 0.4 | 0.13 | 0 | 0.09 | 0.21 | 0.16 | 2.34 | 0.28 | 1.05 | 0 | 0.18 | 0.12 | 0.51 |
| Gratiot | 7.4 | 1.2 | 0.41 | 0.02 | 0.29 | 0.55 | 0.37 | 5.29 | 0.63 | 3.68 | 0.28 | 1.25 | 1.01 | 1.93 |
| Hillsdale | 5.3 | 0.7 | 0.25 | 0.04 | 0.16 | 0.35 | 0.26 | 3.73 | 0.44 | 2.74 | 0.31 | 0.73 | 0.82 | 1.22 |
| Houghton | 1.7 | 0.5 | 0.16 | 0.01 | 0.11 | 0.19 | 0.10 | 1.38 | 0.17 | 1.16 | 0.17 | 0.34 | 0.39 | 0.52 |
| Huron | 4.5 | 0.6 | 0.23 | 0.05 | 0.14 | 0.31 | 0.22 | 3.14 | 0.37 | 2.33 | 0.32 | 0.81 | 0.72 | 1.16 |
| Ingham | 5.0 | 0.7 | 0.25 | 0.03 | 0.16 | 0.36 | 0.25 | 3.52 | 0.42 | 1.74 | 0.06 | 0.45 | 0.28 | 0.93 |
| Ionia | 6.8 | 1.1 | 0.39 | 0.05 | 0.25 | 0.49 | 0.34 | 4.87 | 0.58 | 3.16 | 0.19 | 1.46 | 0.81 | 2.21 |
| Iosco | 2.9 | 0.4 | 0.18 | 0.05 | 0.1 | 0.22 | 0.14 | 2.06 | 0.24 | 1.30 | 0.06 | 0.27 | 0.32 | 0.55 |
| Iron | 1.6 | 0.2 | 0.07 | 0 | 0.05 | 0.11 | 0.08 | 1.14 | 0.13 | 0.83 | 0.08 | 0.2 | 0.24 | 0.32 |
| Isabella | 5.5 | 1.0 | 0.39 | 0.07 | 0.23 | 0.49 | 0.29 | 4.17 | 0.49 | 2.32 | 0.05 | 0.81 | 0.5 | 1.48 |
| Jackson | 5.9 | 0.8 | 0.33 | 0.07 | 0.19 | 0.4 | 0.29 | 4.11 | 0.49 | 2.81 | 0.16 | 0.91 | 0.77 | 1.54 |
| Kalamazoo | 8.9 | 1.5 | 0.57 | 0.07 | 0.36 | 0.71 | 0.45 | 6.48 | 0.77 | 3.22 | 0.11 | 1.39 | 0.52 | 2.07 |
| Kalkaska | 2.7 | 0.4 | 0.15 | 0.03 | 0.09 | 0.2 | 0.14 | 1.94 | 0.23 | 1.05 | 0.02 | 0.28 | 0.2 | 0.56 |
| Kent | 5.7 | 0.6 | 0.24 | 0.04 | 0.15 | 0.37 | 0.27 | 3.84 | 0.45 | 2.24 | 0.14 | 0.97 | 0.5 | 1.41 |
| Keweenaw | 1.5 | 0.5 | 0.16 | 0.02 | 0.11 | 0.17 | 0.09 | 1.23 | 0.15 | 1.18 | 0.27 | 0.22 | 0.43 | 0.33 |
| Lake | 2.7 | 0.3 | 0.15 | 0.05 | 0.08 | 0.2 | 0.12 | 1.79 | 0.21 | 1.24 | 0.05 | 0.6 | 0.32 | 0.93 |
| Lapeer | 5.1 | 0.7 | 0.25 | 0.02 | 0.17 | 0.33 | 0.25 | 3.55 | 0.42 | 1.93 | 0.05 | 0.41 | 0.4 | 0.94 |
| Leelanau | 3.3 | 0.4 | 0.12 | 0 | 0.09 | 0.2 | 0.16 | 2.30 | 0.27 | 1.05 | 0 | 0.18 | 0.15 | 0.52 |
| Lenawee | 7.1 | 1.1 | 0.42 | 0.07 | 0.26 | 0.48 | 0.36 | 5.15 | 0.61 | 3.41 | 0.31 | 1.17 | 0.93 | 1.89 |
| Livingston | 4.7 | 0.6 | 0.21 | 0 | 0.16 | 0.27 | 0.23 | 3.34 | 0.40 | 1.34 | 0 | 0.2 | 0.12 | 0.68 |
| Luce | 0.8 | 0.1 | 0.07 | 0.02 | 0.03 | 0.07 | 0.04 | 0.57 | 0.07 | 0.43 | 0.05 | 0.15 | 0.13 | 0.21 |
| Mackinac | 0.6 | 0.1 | 0.03 | 0 | 0.02 | 0.04 | 0.03 | 0.40 | 0.05 | 0.25 | 0.01 | 0.06 | 0.06 | 0.11 |
| Macomb | 7.2 | 1.2 | 0.44 | 0.05 | 0.29 | 0.49 | 0.36 | 5.14 | 0.61 | 2.52 | 0.03 | 0.66 | 0.4 | 1.41 |
| Manistee | 1.8 | 0.2 | 0.09 | 0.02 | 0.05 | 0.12 | 0.09 | 1.24 | 0.15 | 0.89 | 0.08 | 0.34 | 0.26 | 0.52 |
| Marquette | 2.0 | 0.4 | 0.14 | 0.01 | 0.1 | 0.17 | 0.11 | 1.52 | 0.18 | 0.91 | 0.03 | 0.21 | 0.22 | 0.44 |
| Mason | 2.1 | 0.3 | 0.12 | 0.02 | 0.08 | 0.16 | 0.10 | 1.50 | 0.18 | 1.03 | 0.07 | 0.33 | 0.29 | 0.52 |
| Mecosta | 3.7 | 0.6 | 0.21 | 0.02 | 0.14 | 0.29 | 0.19 | 2.70 | 0.32 | 1.61 | 0.04 | 0.39 | 0.38 | 0.78 |
| Menominee | 2.8 | 0.4 | 0.1 | 0.01 | 0.09 | 0.19 | 0.15 | 2.19 | 0.26 | 1.53 | 0.1 | 0.48 | 0.42 | 0.83 |
| Midland | 6.8 | 1.1 | 0.4 | 0.04 | 0.27 | 0.54 | 0.34 | 4.88 | 0.58 | 2.31 | 0.02 | 0.63 | 0.32 | 1.31 |
| Missaukee | 2.5 | 0.3 | 0.14 | 0.04 | 0.08 | 0.18 | 0.12 | 1.75 | 0.21 | 1.13 | 0.04 | 0.33 | 0.29 | 0.54 |
| Monroe | 9.5 | 2.0 | 0.67 | 0.08 | 0.47 | 0.77 | 0.51 | 7.22 | 0.86 | 4.46 | 0.19 | 0.89 | 1.1 | 1.98 |
| Montcalm | 5.9 | 0.9 | 0.33 | 0.06 | 0.21 | 0.43 | 0.29 | 4.21 | 0.50 | 2.80 | 0.16 | 1.2 | 0.76 | 1.8 |
| Montmorency | 3.6 | 0.6 | 0.28 | 0.06 | 0.16 | 0.29 | 0.18 | 2.64 | 0.31 | 1.67 | 0.06 | 0.58 | 0.43 | 0.97 |
| Muskegon | 1.3 | 0.0 | 0.02 | 0.01 | 0.01 | 0.07 | 0.06 | 0.83 | 0.10 | 0.50 | 0.02 | 0.18 | 0.11 | 0.28 |
| Newaygo | 2.9 | 0.4 | 0.16 | 0.03 | 0.09 | 0.22 | 0.14 | 2.07 | 0.24 | 1.23 | 0.03 | 0.35 | 0.28 | 0.72 |
| Oakland | 6.5 | 1.0 | 0.34 | 0.03 | 0.24 | 0.4 | 0.31 | 4.49 | 0.53 | 1.85 | 0.04 | 0.57 | 0.17 | 1.11 |
| Oceana | 1.6 | 0.1 | 0.05 | 0.01 | 0.03 | 0.1 | 0.07 | 1.01 | 0.12 | 0.68 | 0.04 | 0.22 | 0.18 | 0.37 |
| Ogemaw | 4.0 | 0.8 | 0.34 | 0.08 | 0.19 | 0.35 | 0.21 | 3.00 | 0.36 | 1.96 | 0.09 | 0.52 | 0.51 | 0.89 |
| Ontonagon | 2.1 | 0.3 | 0.09 | 0.01 | 0.07 | 0.15 | 0.09 | 1.35 | 0.16 | 0.99 | 0.09 | 0.14 | 0.31 | 0.23 |
| Osceola | 3.0 | 0.5 | 0.17 | 0.03 | 0.11 | 0.23 | 0.15 | 2.13 | 0.25 | 1.38 | 0.05 | 0.38 | 0.35 | 0.66 |
| Oscoda | 3.5 | 0.6 | 0.26 | 0.06 | 0.15 | 0.29 | 0.18 | 2.57 | 0.31 | 1.66 | 0.09 | 0.53 | 0.44 | 0.83 |
| Otsego | 3.2 | 0.4 | 0.17 | 0.05 | 0.1 | 0.22 | 0.15 | 2.17 | 0.26 | 1.12 | 0 | 0.27 | 0.19 | 0.57 |
| Ottawa | 3.9 | 0.3 | 0.12 | 0.02 | 0.07 | 0.22 | 0.18 | 2.63 | 0.31 | 1.32 | 0.03 | 0.36 | 0.26 | 0.92 |
| Presque Isle | 2.8 | 0.5 | 0.18 | 0.02 | 0.12 | 0.22 | 0.14 | 2.04 | 0.24 | 1.33 | 0.07 | 0.4 | 0.36 | 0.68 |
| Roscommon | 3.2 | 0.7 | 0.28 | 0.06 | 0.15 | 0.28 | 0.17 | 2.45 | 0.29 | 1.38 | 0.01 | 0.26 | 0.31 | 0.53 |
| Saginaw | 6.8 | 1.1 | 0.44 | 0.08 | 0.26 | 0.53 | 0.34 | 4.86 | 0.58 | 3.20 | 0.28 | 1.49 | 0.7 | 2.06 |
| St. Clair | 6.1 | 1.1 | 0.41 | 0.07 | 0.25 | 0.45 | 0.31 | 4.41 | 0.52 | 2.82 | 0.2 | 1.12 | 0.72 | 1.73 |
| St. Joseph | 7.9 | 1.2 | 0.5 | 0.12 | 0.28 | 0.57 | 0.39 | 5.65 | 0.67 | 4.04 | 0.4 | 1.25 | 1.15 | 2 |
| Sanilac | 4.7 | 0.7 | 0.28 | 0.04 | 0.17 | 0.34 | 0.23 | 3.36 | 0.40 | 2.41 | 0.27 | 0.89 | 0.71 | 1.34 |
| Schoolcraft | 0.5 | 0.0 | 0.01 | 0 | 0.01 | 0.03 | 0.03 | 0.37 | 0.04 | 0.26 | 0.03 | 0.13 | 0.07 | 0.16 |
| Shiawassee | 5.2 | 0.8 | 0.26 | 0.02 | 0.18 | 0.35 | 0.26 | 3.66 | 0.43 | 2.18 | 0.13 | 0.27 | 0.51 | 0.76 |
| Tuscola | 6.1 | 1.0 | 0.38 | 0.06 | 0.24 | 0.45 | 0.31 | 4.37 | 0.52 | 2.84 | 0.16 | 0.84 | 0.74 | 1.48 |
| Van Buren | 6.9 | 1.0 | 0.43 | 0.12 | 0.23 | 0.51 | 0.35 | 4.98 | 0.59 | 3.24 | 0.24 | 1.37 | 0.75 | 1.95 |
| Washtenaw | 7.0 | 1.0 | 0.37 | 0.03 | 0.24 | 0.45 | 0.35 | 5.04 | 0.60 | 2.05 | 0 | 0.39 | 0.18 | 1.13 |
| Wayne | 7.9 | 1.2 | 0.57 | 0.16 | 0.3 | 0.63 | 0.40 | 5.67 | 0.67 | 4.16 | 0.35 | 1.72 | 0.75 | 2.5 |
| Wexford | 2.2 | 0.2 | 0.1 | 0.03 | 0.05 | 0.14 | 0.11 | 1.53 | 0.18 | 0.97 | 0.03 | 0.23 | 0.25 | 0.45 |

Table A4. Extreme heat (EH) days and attributable fraction days (AFDs) in the projected period (2041-2070) for mortality, emergency department (ED) visits, and renal, respiratory and heat-related hospital admissions (HA).

|  | EH days | | Mortality AFDs | | | | ED visit AFDs | | | HA AFDs | | | | |
| --- | --- | --- | --- | --- | --- | --- | --- | --- | --- | --- | --- | --- | --- | --- |
| County | 32.3-34.9 °C | 35 °C | men ages 20-54 | women ages 20-54 | ages 55-64 | ages 65+ | ages 0-17 | ages 18-64 (heat-related only) | ages 65+ | non-whites, ages 0-64 | ages 65-77, nonblack | ages 65-77, black | ages 78+, nonblack | ages 78+, black |
| Alcona | 12.7 | 5.6 | 2.04 | 0.21 | 1.35 | 1.75 | 0.83 | 11.69 | 1.41 | 1.14 | 0.33 | 1.44 | 1.67 | 3.01 |
| Alger | 9.7 | 5.9 | 1.94 | 0.06 | 1.39 | 1.58 | 0.73 | 10.19 | 1.24 | 0.77 | 0.07 | 1.56 | 1.19 | 2.92 |
| Allegan | 19.1 | 6.6 | 2.24 | 0.13 | 1.64 | 1.78 | 1.15 | 16.22 | 1.95 | 2.36 | 0.37 | 2.82 | 2.31 | 4.42 |
| Alpena | 12.0 | 7.4 | 2.44 | 0.06 | 1.77 | 1.94 | 0.89 | 12.39 | 1.50 | 1.18 | 0.16 | 2.09 | 1.89 | 3.66 |
| Antrim | 14.7 | 4.5 | 1.49 | 0.1 | 1.05 | 1.41 | 0.85 | 12.08 | 1.45 | 1.07 | 0.11 | 1.23 | 1.25 | 2.8 |
| Arenac | 14.7 | 8.2 | 2.94 | 0.26 | 1.97 | 2.34 | 1.07 | 14.94 | 1.81 | 1.87 | 0.46 | 3.49 | 2.41 | 5.4 |
| Baraga | 10.5 | 5.4 | 1.84 | 0.07 | 1.31 | 1.49 | 0.74 | 10.35 | 1.25 | 1.33 | 0.67 | 2.39 | 2.22 | 3.82 |
| Barry | 21.2 | 9.3 | 3.19 | 0.22 | 2.23 | 2.38 | 1.36 | 19.19 | 2.31 | 2.84 | 0.41 | 3.88 | 2.83 | 6.4 |
| Bay | 18.9 | 9.8 | 3.23 | 0.11 | 2.34 | 2.62 | 1.31 | 18.42 | 2.23 | 2.61 | 0.6 | 2.92 | 2.56 | 5.52 |
| Benzie | 13.5 | 4.6 | 1.52 | 0.01 | 1.05 | 1.3 | 0.82 | 11.65 | 1.40 | 0.81 | 0.03 | 1.01 | 0.78 | 2.69 |
| Berrien | 23.1 | 9.9 | 3.51 | 0.4 | 2.34 | 2.94 | 1.49 | 20.95 | 2.52 | 3.81 | 0.7 | 5.27 | 3.14 | 7.65 |
| Branch | 20.1 | 7.5 | 2.68 | 0.28 | 1.81 | 2.23 | 1.23 | 17.38 | 2.09 | 3.08 | 1.62 | 3.69 | 3.95 | 5.86 |
| Calhoun | 20.8 | 8.9 | 3.11 | 0.24 | 2.15 | 2.58 | 1.34 | 18.80 | 2.26 | 2.90 | 0.69 | 3.78 | 3.13 | 6.11 |
| Cass | 23.4 | 10.3 | 3.69 | 0.42 | 2.46 | 3.03 | 1.51 | 21.25 | 2.56 | 3.99 | 1.04 | 4.29 | 3.94 | 6.97 |
| Charlevoix | 13.3 | 3.5 | 1.23 | 0.08 | 0.93 | 1.18 | 0.74 | 10.54 | 1.26 | 0.94 | 0.07 | 1.42 | 1.3 | 2.69 |
| Cheboygan | 12.5 | 4.1 | 1.39 | 0.06 | 1.05 | 1.37 | 0.72 | 10.25 | 1.23 | 0.92 | 0.12 | 1.92 | 1.41 | 3.43 |
| Chippewa | 6.0 | 2.3 | 0.79 | 0.07 | 0.53 | 0.74 | 0.38 | 5.31 | 0.64 | 0.28 | 0.2 | 0.8 | 0.88 | 1.79 |
| Clare | 16.0 | 8.1 | 2.93 | 0.36 | 1.93 | 2.53 | 1.10 | 15.40 | 1.86 | 2.00 | 0.12 | 2.39 | 2.2 | 4.34 |
| Clinton | 19.4 | 8.4 | 2.76 | 0.02 | 2.05 | 2.53 | 1.25 | 17.56 | 2.12 | 2.20 | 0.47 | 2.39 | 1.84 | 4.42 |
| Crawford | 12.7 | 6.4 | 2.24 | 0.2 | 1.55 | 1.85 | 0.86 | 12.10 | 1.46 | 1.13 | 0.01 | 1.59 | 1.33 | 3.25 |
| Delta | 6.3 | 1.5 | 0.52 | 0.01 | 0.38 | 0.57 | 0.36 | 5.07 | 0.60 | 0.28 | 0.16 | 0.96 | 0.88 | 1.53 |
| Dickinson | 11.6 | 5.5 | 1.74 | 0.01 | 1.26 | 1.48 | 0.77 | 10.79 | 1.30 | 1.25 | 0.24 | 2.04 | 1.74 | 3.4 |
| Eaton | 18.8 | 8.3 | 2.72 | 0.07 | 2 | 2.21 | 1.22 | 17.22 | 2.08 | 2.06 | 0.48 | 1.49 | 1.96 | 3.75 |
| Emmet | 10.3 | 2.4 | 0.8 | 0.01 | 0.61 | 0.84 | 0.56 | 7.96 | 0.95 | 0.50 | 0.02 | 0.65 | 0.82 | 1.53 |
| Genesee | 17.5 | 7.8 | 2.65 | 0.15 | 1.88 | 2.34 | 1.14 | 16.04 | 1.93 | 1.91 | 0.2 | 3.63 | 1.42 | 5.4 |
| Gladwin | 17.2 | 9.4 | 3.29 | 0.27 | 2.26 | 2.76 | 1.23 | 17.21 | 2.08 | 2.36 | 0.18 | 2.29 | 2.4 | 4.65 |
| Gogebic | 7.7 | 2.1 | 0.67 | 0.02 | 0.5 | 0.81 | 0.43 | 6.13 | 0.73 | 0.58 | 0.65 | 1.07 | 1.53 | 1.82 |
| Grand Traverse | 15.1 | 6.2 | 1.95 | 0.02 | 1.44 | 1.6 | 0.95 | 13.40 | 1.61 | 1.05 | 0.03 | 1.06 | 0.66 | 2.87 |
| Gratiot | 21.7 | 10.2 | 3.33 | 0.07 | 2.44 | 2.8 | 1.43 | 20.17 | 2.43 | 3.68 | 1.04 | 4.56 | 3.77 | 7.02 |
| Hillsdale | 19.5 | 6.8 | 2.31 | 0.13 | 1.64 | 2 | 1.18 | 16.64 | 2.00 | 2.74 | 1.32 | 3.22 | 3.54 | 5.4 |
| Houghton | 8.2 | 4.9 | 1.62 | 0.06 | 1.17 | 1.48 | 0.60 | 8.42 | 1.02 | 1.16 | 1.04 | 2.01 | 2.35 | 3.11 |
| Huron | 18.0 | 7.3 | 2.53 | 0.19 | 1.76 | 2.17 | 1.14 | 16.06 | 1.93 | 2.33 | 1.57 | 3.98 | 3.6 | 5.76 |
| Ingham | 18.3 | 7.6 | 2.54 | 0.1 | 1.82 | 2.34 | 1.16 | 16.40 | 1.97 | 1.74 | 0.28 | 2.05 | 1.28 | 4.21 |
| Ionia | 20.5 | 9.3 | 3.09 | 0.14 | 2.22 | 2.43 | 1.34 | 18.85 | 2.27 | 3.16 | 0.68 | 4.74 | 3 | 7.2 |
| Iosco | 13.4 | 6.3 | 2.17 | 0.21 | 1.48 | 1.87 | 0.88 | 12.43 | 1.50 | 1.30 | 0.35 | 1.62 | 1.87 | 3.29 |
| Iron | 8.5 | 3.3 | 1.03 | 0.02 | 0.79 | 1.02 | 0.53 | 7.43 | 0.89 | 0.83 | 0.5 | 1.36 | 1.53 | 2.21 |
| Isabella | 18.5 | 9.0 | 3.11 | 0.24 | 2.1 | 2.97 | 1.26 | 17.72 | 2.14 | 2.32 | 0.2 | 3.06 | 2.1 | 5.67 |
| Jackson | 21.0 | 8.5 | 3 | 0.26 | 2.05 | 2.42 | 1.31 | 18.54 | 2.23 | 2.81 | 0.7 | 3.95 | 3.37 | 6.64 |
| Kalamazoo | 24.0 | 11.6 | 3.94 | 0.18 | 2.78 | 3.39 | 1.62 | 22.74 | 2.75 | 3.22 | 0.36 | 4.36 | 1.78 | 6.68 |
| Kalkaska | 13.3 | 5.5 | 1.86 | 0.14 | 1.29 | 1.67 | 0.85 | 12.04 | 1.45 | 1.05 | 0.12 | 1.59 | 1.23 | 3.23 |
| Kent | 19.3 | 7.0 | 2.33 | 0.13 | 1.67 | 2.04 | 1.16 | 16.43 | 1.97 | 2.24 | 0.61 | 4.08 | 2.08 | 5.79 |
| Keweenaw | 7.3 | 4.7 | 1.57 | 0.09 | 1.14 | 1.39 | 0.55 | 7.74 | 0.94 | 1.18 | 1.67 | 1.45 | 2.65 | 2.23 |
| Lake | 13.1 | 5.1 | 1.85 | 0.22 | 1.24 | 1.77 | 0.79 | 11.15 | 1.34 | 1.24 | 0.3 | 3.49 | 1.91 | 5.38 |
| Lapeer | 19.4 | 7.8 | 2.55 | 0.07 | 1.87 | 2 | 1.21 | 17.08 | 2.06 | 1.93 | 0.26 | 1.94 | 1.86 | 4.4 |
| Leelanau | 15.3 | 5.8 | 1.88 | 0 | 1.4 | 1.51 | 0.95 | 13.41 | 1.61 | 1.05 | 0 | 1.02 | 0.86 | 2.9 |
| Lenawee | 22.7 | 9.9 | 3.4 | 0.23 | 2.36 | 2.5 | 1.48 | 20.84 | 2.51 | 3.41 | 1.19 | 4.56 | 3.64 | 7.42 |
| Livingston | 17.7 | 7.2 | 2.32 | 0 | 1.72 | 1.48 | 1.12 | 15.75 | 1.90 | 1.34 | 0.02 | 0.92 | 0.57 | 3.14 |
| Luce | 7.5 | 2.6 | 1.08 | 0.23 | 0.62 | 0.93 | 0.45 | 6.40 | 0.77 | 0.43 | 0.53 | 1.62 | 1.42 | 2.38 |
| Mackinac | 6.4 | 1.8 | 0.59 | 0.04 | 0.47 | 0.63 | 0.36 | 5.11 | 0.61 | 0.25 | 0.08 | 0.84 | 0.74 | 1.5 |
| Macomb | 21.4 | 10.6 | 3.54 | 0.14 | 2.53 | 2.34 | 1.44 | 20.17 | 2.44 | 2.52 | 0.13 | 2.51 | 1.5 | 5.32 |
| Manistee | 10.6 | 3.7 | 1.33 | 0.11 | 0.92 | 1.18 | 0.63 | 8.96 | 1.08 | 0.89 | 0.54 | 2.23 | 1.82 | 3.44 |
| Marquette | 10.0 | 5.7 | 1.87 | 0.05 | 1.38 | 1.52 | 0.73 | 10.19 | 1.24 | 0.91 | 0.17 | 1.38 | 1.42 | 2.87 |
| Mason | 10.6 | 4.6 | 1.55 | 0.08 | 1.11 | 1.34 | 0.68 | 9.58 | 1.15 | 1.03 | 0.47 | 2.11 | 1.8 | 3.32 |
| Mecosta | 15.0 | 6.5 | 2.19 | 0.1 | 1.56 | 2.07 | 0.97 | 13.67 | 1.65 | 1.61 | 0.21 | 1.87 | 1.9 | 3.73 |
| Menominee | 13.5 | 4.8 | 1.41 | 0.05 | 1.17 | 1.54 | 0.89 | 12.53 | 1.51 | 1.53 | 0.55 | 2.63 | 2.36 | 4.46 |
| Midland | 20.3 | 10.2 | 3.36 | 0.12 | 2.44 | 2.94 | 1.37 | 19.27 | 2.33 | 2.31 | 0.09 | 2.36 | 1.25 | 4.94 |
| Missaukee | 12.1 | 4.7 | 1.69 | 0.2 | 1.13 | 1.47 | 0.75 | 10.64 | 1.28 | 1.13 | 0.25 | 1.96 | 1.73 | 3.23 |
| Monroe | 24.8 | 14.1 | 4.52 | 0.2 | 3.32 | 3.41 | 1.78 | 24.92 | 3.02 | 4.46 | 0.64 | 3.1 | 3.63 | 6.76 |
| Montcalm | 18.7 | 8.4 | 2.85 | 0.19 | 2 | 2.45 | 1.22 | 17.19 | 2.07 | 2.80 | 0.63 | 4.4 | 3 | 6.64 |
| Montmorency | 14.7 | 8.0 | 2.9 | 0.24 | 1.94 | 2.27 | 1.04 | 14.54 | 1.76 | 1.67 | 0.34 | 2.88 | 2.31 | 4.88 |
| Muskegon | 8.8 | 2.0 | 0.69 | 0.08 | 0.47 | 0.78 | 0.47 | 6.71 | 0.80 | 0.50 | 0.12 | 1.38 | 0.85 | 2.22 |
| Newaygo | 13.3 | 5.3 | 1.82 | 0.13 | 1.26 | 1.74 | 0.83 | 11.74 | 1.41 | 1.23 | 0.2 | 1.81 | 1.65 | 3.64 |
| Oakland | 21.3 | 9.5 | 3.12 | 0.08 | 2.27 | 1.98 | 1.36 | 19.12 | 2.30 | 1.85 | 0.16 | 2.34 | 0.69 | 4.51 |
| Oceana | 10.6 | 3.0 | 1 | 0.09 | 0.69 | 1.1 | 0.59 | 8.32 | 0.99 | 0.68 | 0.29 | 1.67 | 1.44 | 2.79 |
| Ogemaw | 15.0 | 7.9 | 2.87 | 0.31 | 1.91 | 2.42 | 1.04 | 14.62 | 1.77 | 1.96 | 0.45 | 2.6 | 2.47 | 4.43 |
| Ontonagon | 9.8 | 3.8 | 1.14 | 0.04 | 0.87 | 1.16 | 0.57 | 7.98 | 0.96 | 0.99 | 0.51 | 1.04 | 1.72 | 1.72 |
| Osceola | 13.5 | 5.4 | 1.85 | 0.12 | 1.31 | 1.71 | 0.85 | 11.96 | 1.44 | 1.38 | 0.27 | 2.07 | 1.91 | 3.63 |
| Oscoda | 14.5 | 7.3 | 2.57 | 0.25 | 1.74 | 2.2 | 0.99 | 13.84 | 1.67 | 1.66 | 0.49 | 2.75 | 2.3 | 4.38 |
| Otsego | 14.3 | 5.7 | 1.99 | 0.22 | 1.37 | 1.69 | 0.89 | 12.51 | 1.50 | 1.12 | 0.01 | 1.52 | 1.07 | 3.21 |
| Ottawa | 15.0 | 3.9 | 1.31 | 0.08 | 0.91 | 1.14 | 0.85 | 12.02 | 1.44 | 1.32 | 0.11 | 1.44 | 1.07 | 3.68 |
| Presque Isle | 12.6 | 7.5 | 2.47 | 0.09 | 1.8 | 2 | 0.92 | 12.87 | 1.56 | 1.33 | 0.4 | 2.35 | 2.19 | 4.03 |
| Roscommon | 13.0 | 6.8 | 2.45 | 0.26 | 1.61 | 2.03 | 0.91 | 12.75 | 1.54 | 1.38 | 0.04 | 1.37 | 1.57 | 2.88 |
| Saginaw | 21.0 | 10.3 | 3.55 | 0.25 | 2.46 | 3.08 | 1.41 | 19.85 | 2.40 | 3.20 | 1.11 | 5.87 | 2.81 | 8.1 |
| St. Clair | 21.2 | 10.8 | 3.72 | 0.25 | 2.59 | 2.74 | 1.43 | 20.11 | 2.43 | 2.82 | 0.9 | 5.1 | 3.24 | 7.87 |
| St. Joseph | 23.2 | 10.0 | 3.58 | 0.35 | 2.42 | 2.92 | 1.50 | 21.06 | 2.54 | 4.04 | 1.46 | 4.56 | 4.21 | 7.27 |
| Sanilac | 18.8 | 7.9 | 2.76 | 0.17 | 1.9 | 2.29 | 1.21 | 17.05 | 2.05 | 2.41 | 1.35 | 4.25 | 3.54 | 6.38 |
| Schoolcraft | 5.9 | 1.7 | 0.55 | 0.05 | 0.45 | 0.61 | 0.35 | 4.93 | 0.59 | 0.26 | 0.3 | 1.42 | 0.98 | 1.91 |
| Shiawassee | 18.3 | 7.5 | 2.45 | 0.06 | 1.78 | 1.97 | 1.15 | 16.27 | 1.96 | 2.18 | 0.52 | 1.17 | 2.16 | 3.38 |
| Tuscola | 20.3 | 9.6 | 3.27 | 0.2 | 2.3 | 2.55 | 1.35 | 18.95 | 2.29 | 2.84 | 0.7 | 3.54 | 3.13 | 6.21 |
| Van Buren | 21.5 | 8.4 | 3.07 | 0.36 | 2 | 2.71 | 1.36 | 19.22 | 2.31 | 3.24 | 0.96 | 5.08 | 2.92 | 7.23 |
| Washtenaw | 22.1 | 9.6 | 3.25 | 0.1 | 2.33 | 2.25 | 1.44 | 20.20 | 2.43 | 2.05 | 0.02 | 1.44 | 0.71 | 4.25 |
| Wayne | 23.9 | 11.1 | 4.06 | 0.47 | 2.67 | 3.5 | 1.58 | 22.17 | 2.67 | 4.16 | 1.34 | 6.52 | 2.85 | 9.49 |
| Wexford | 11.9 | 3.9 | 1.46 | 0.17 | 0.95 | 1.28 | 0.71 | 10.06 | 1.21 | 0.97 | 0.21 | 1.49 | 1.59 | 2.94 |

Table A5. Annual population (ages 0-99) and extreme-heat (EH) associated mortality and emergency department (ED) visit rates (per 100,000 population) by county and historical (1971-2000) or projected (2041-2070) time period.

| County | Historical Population (2000) | Projected Population (2050) [105] | Historical EH Mortality Rates | Projected EH Mortality Rates | Historical EH ED Rates | Projected EH ED Rates |
| --- | --- | --- | --- | --- | --- | --- |
| Alcona | 11440 | 4518 | 0.29 | 2.89 | 7.9 | 52.0 |
| Alger | 9775 | 4754 | 0.24 | 2.7 | 4.7 | 44.3 |
| Allegan | 111420 | 152941 | 0.33 | 2.63 | 10.7 | 64.7 |
| Alpena | 30278 | 11100 | 0.3 | 2.4 | 6.3 | 44.8 |
| Antrim | 24296 | 17154 | 0.18 | 2.25 | 6.7 | 54.0 |
| Arenac | 16869 | 5658 | 0.45 | 3.61 | 10.0 | 60.8 |
| Baraga | 8950 | 4925 | 0.3 | 1.87 | 6.0 | 38.2 |
| Barry | 60212 | 50300 | 0.44 | 3.27 | 13.1 | 72.2 |
| Bay | 108264 | 81585 | 0.53 | 2.96 | 13.6 | 65.0 |
| Benzie | 17913 | 11411 | 0.17 | 2.11 | 6.4 | 55.1 |
| Berrien | 157371 | 145115 | 0.76 | 3.49 | 17.9 | 73.6 |
| Branch | 47279 | 27077 | 0.43 | 2.72 | 11.5 | 63.3 |
| Calhoun | 137652 | 115343 | 0.54 | 2.59 | 12.9 | 61.3 |
| Cass | 52741 | 23942 | 0.64 | 3.16 | 17.2 | 71.1 |
| Charlevoix | 26605 | 18863 | 0.13 | 1.67 | 5.2 | 45.0 |
| Cheboygan | 27317 | 16600 | 0.16 | 1.7 | 5.1 | 41.3 |
| Chippewa | 39336 | 33029 | 0.04 | 0.94 | 1.3 | 21.8 |
| Clare | 31370 | 18384 | 0.55 | 3.44 | 11.1 | 57.0 |
| Clinton | 73731 | 54121 | 0.34 | 2.49 | 11.2 | 60.9 |
| Crawford | 14460 | 6866 | 0.3 | 2.86 | 6.7 | 52.3 |
| Delta | 37787 | 19766 | 0.04 | 0.53 | 1.5 | 16.6 |
| Dickinson | 26966 | 12933 | 0.29 | 1.18 | 6.7 | 32.3 |
| Eaton | 108271 | 104287 | 0.34 | 2.59 | 10.2 | 62.3 |
| Emmet | 32876 | 29032 | 0.07 | 1.14 | 2.9 | 34.4 |
| Genesee | 439038 | 492163 | 0.38 | 2.74 | 9.6 | 55.5 |
| Gladwin | 26281 | 13481 | 0.57 | 3.41 | 14.1 | 64.8 |
| Gogebic | 16548 | 4503 | 0.13 | 0.58 | 2.6 | 17.9 |
| Grand Traverse | 85608 | 108671 | 0.21 | 2.51 | 6.7 | 58.6 |
| Gratiot | 42829 | 22262 | 0.72 | 2.83 | 15.5 | 67.5 |
| Hillsdale | 47444 | 25932 | 0.37 | 1.97 | 11.2 | 56.1 |
| Houghton | 35847 | 26574 | 0.28 | 1.51 | 4.2 | 30.8 |
| Huron | 34096 | 13149 | 0.44 | 2.61 | 11.3 | 58.7 |
| Ingham | 281815 | 421950 | 0.29 | 2.51 | 8.6 | 59.9 |
| Ionia | 65362 | 54057 | 0.44 | 2.97 | 12.2 | 71.1 |
| Iosco | 26887 | 7282 | 0.37 | 2.63 | 7.9 | 46.0 |
| Iron | 12123 | 4074 | 0.22 | 0.99 | 4.5 | 24.2 |
| Isabella | 69063 | 88892 | 0.39 | 2.63 | 10.0 | 65.3 |
| Jackson | 163405 | 155839 | 0.47 | 2.94 | 11.6 | 65.7 |
| Kalamazoo | 245034 | 339634 | 0.66 | 3.59 | 17.4 | 84.6 |
| Kalkaska | 17548 | 10852 | 0.21 | 3.04 | 5.7 | 50.7 |
| Kent | 597478 | 1012096 | 0.32 | 2.41 | 10.2 | 60.6 |
| Keweenaw | 2033 | 1863 | 0.26 | 2.27 | 4.4 | 33.7 |
| Lake | 11698 | 4504 | 0.29 | 2.53 | 6.3 | 45.3 |
| Lapeer | 91397 | 95921 | 0.27 | 2.94 | 9.5 | 67.4 |
| Leelanau | 21867 | 17827 | 0.18 | 2.51 | 8.1 | 62.2 |
| Lenawee | 101890 | 88652 | 0.51 | 2.96 | 14.8 | 76.2 |
| Livingston | 182186 | 330569 | 0.18 | 2.43 | 8.6 | 67.0 |
| Luce | 6810 | 4528 | 0.08 | 1.43 | 1.7 | 27.9 |
| Mackinac | 11485 | 4615 | 0.05 | 0.83 | 1.4 | 19.3 |
| Macomb | 835199 | 1098046 | 0.53 | 3.2 | 14.8 | 78.6 |
| Manistee | 25138 | 9063 | 0.15 | 1.69 | 4.2 | 38.0 |
| Marquette | 66099 | 43159 | 0.19 | 1.43 | 4.3 | 35.3 |
| Mason | 28698 | 11680 | 0.2 | 1.65 | 5.0 | 35.9 |
| Mecosta | 42666 | 27440 | 0.27 | 1.7 | 7.9 | 48.8 |
| Menominee | 24387 | 8413 | 0.25 | 1.21 | 7.2 | 37.7 |
| Midland | 83660 | 72273 | 0.47 | 3.02 | 14.4 | 68.7 |
| Missaukee | 15041 | 7414 | 0.2 | 2.02 | 5.5 | 42.8 |
| Monroe | 153578 | 172683 | 0.74 | 4.65 | 19.9 | 97.2 |
| Montcalm | 63699 | 61234 | 0.45 | 3.11 | 12.1 | 64.9 |
| Montmorency | 9953 | 4973 | 0.49 | 3.37 | 10.5 | 61.6 |
| Muskegon | 173923 | 197642 | 0.07 | 0.99 | 2.4 | 24.4 |
| Newaygo | 49450 | 46678 | 0.22 | 2.05 | 6.2 | 42.9 |
| Oakland | 1199174 | 1541022 | 0.35 | 2.6 | 12.3 | 72.1 |
| Oceana | 27358 | 21022 | 0.1 | 1.23 | 3.1 | 30.8 |
| Ogemaw | 22018 | 9148 | 0.61 | 3.89 | 10.3 | 55.9 |
| Ontonagon | 7103 | 4893 | 0.24 | 1.87 | 5.3 | 33.6 |
| Osceola | 23897 | 10626 | 0.25 | 1.78 | 6.7 | 40.1 |
| Oscoda | 9051 | 4277 | 0.41 | 3.25 | 9.4 | 53.4 |
| Otsego | 24772 | 18983 | 0.21 | 2.49 | 6.7 | 52.1 |
| Ottawa | 259811 | 507501 | 0.16 | 1.35 | 7.2 | 48.5 |
| Presque Isle | 13953 | 4443 | 0.3 | 2.39 | 8.0 | 50.4 |
| Roscommon | 25345 | 16456 | 0.45 | 3.46 | 9.5 | 58.8 |
| Saginaw | 203395 | 187966 | 0.67 | 3.03 | 14.6 | 63.6 |
| St. Clair | 167852 | 207028 | 0.48 | 3.95 | 12.6 | 79.6 |
| St. Joseph | 62306 | 46429 | 0.68 | 3.05 | 16.9 | 70.5 |
| Sanilac | 44795 | 28323 | 0.43 | 2.74 | 10.8 | 62.1 |
| Schoolcraft | 8896 | 4287 | 0.04 | 0.86 | 1.2 | 18.3 |
| Shiawassee | 72508 | 41606 | 0.34 | 1.81 | 10.6 | 52.1 |
| Tuscola | 57295 | 35855 | 0.48 | 2.75 | 13.1 | 66.5 |
| Van Buren | 76763 | 74187 | 0.58 | 3.54 | 14.2 | 70.5 |
| Washtenaw | 344832 | 535074 | 0.27 | 2.55 | 11.8 | 81.5 |
| Wayne | 1905976 | 2116976 | 0.73 | 4.27 | 15.9 | 77.0 |
| Wexford | 32693 | 16882 | 0.16 | 1.44 | 4.6 | 36.9 |

## Appendix 6: Loading data into BenMAP

Because BenMAP assigns one population dataset per analysis, the program was run twice for each health outcome using pre-loaded Census 2000 population data for the historical (1971-2000) analysis and imported ICLUS 2050 population data for the projected (2041-2070) analysis. These instructions are for the mortality and ED visit analyses. A similar process was followed for renal hospitalizations, except that 1) incidence information was uploaded without assigning race for the 0-64 age group, 2) for the 0-64 age group the health impact functions were defined only for BLACK, ASIAN and NATAMER (Native American), and the WHITE health impact function was multiplied by a constant B which was set to zero and 3) constant A was set as 1/153 to convert annual May-September rates to daily hospitalization rates.

1. Select ‘United States’ as the setup.
2. Define the pollutants under 'Modify Datasets'.
   1. Pollutants were the attributable fraction days (AFDs), and were defined for each of the age groups and genders included in the analysis due to the way in which exposure data is loaded in BenMAP.
   2. Define the observation type as 'daily' for all pollutants. Note that we have annual estimates of the sum of EH days rather than hourly or daily exposure data.
   3. Define the metric as ‘AFDs’.
3. Choose the pollutants to be included in the analysis.
   1. Under 'Pollutants', click and drag the AFDs for all age/gender categories under 'selected pollutants'.
4. Load ICLUS 2050 population data under ‘Modify datasets’.
   1. These data were obtained in a format ready for loading into BenMAP; no formatting was required.
5. For the morbidity data only, format the calculated all-cause incidence rates for importing into BenMAP. Load calculated all-cause incidence rates under ‘Modify datasets’.
   1. A sample file with the required headings for loading incidence data can be found under ‘Modify datasets’ > ‘Manage Incidence/Prevalence Rates’ > ‘Edit’ > ‘Output sample file’
   2. One .csv file may be created for each age group. For the 0-17 age group:
      1. Define the Endpoint Group as ‘Emergency Room Visits’, the Endpoint as ‘All cause’, Start age as ‘0’ and the End age as ‘17’. Column should correspond to Michigan’s State FIPS code (26) and Row should correspond to the county FIPS codes.
      2. Continue the list of observations for the 65+ age group with the Start age as ‘65’ and the End age as ‘99’.
      3. For the 18-64 age group, define the Endpoint as “Heat-related”, Start age as 18 and End age as 64.
   3. Upload the all-cause and heat-related incidence datasets under ‘Modify datasets’ > ‘Manage Incidence/Prevalence Rates’ > ‘Add’ > ‘Load from file’
6. Format the exposure data for importing into BenMAP.
   1. Create separate excel csv files for AFD datasets for each age/gender group.
      1. BenMAP requires data to be loaded with the following 6 headings: Column, Row, Metric, Seasonal Metric, Annual Metric, Values.
         - 1. State FIPS codes must be entered under 'Column' and County FIPS codes must be entered under 'Row'.
           2. Seasonal metric is non-applicable and can be left blank.
           3. Annual metric was defined as ‘sum’, since our values represent the sum annual AFDs for each period.
7. Load the historical AFD datasets under 'baseline' for each age group's 'Source of Air Quality Data' section; load a dataset of zeros under 'control'. Ensure that ‘county’ is selected as grid type.
8. Define the health impact functions under 'Modify Datasets' > ‘Add’.
   1. Separate functions must be defined in BenMAP; one within each age/gender group for each AFD:
      1. Health endpoint was set to ‘Emergency Room Visits, All Cause” for ages 0-17 and 65+ and “Emergency Room Visits, Heat-related” for ages 18-64 or “Mortality, Non-accidental.”
      2. Annual Statistic was set to ‘Sum’

This step ensures that BenMAP considers the exposure data as an annual metric, rather than daily or hourly.

- - 1. For ED visits, constant ‘A’ was defined as the annual-to-warm season incidence rate conversion factor (0.66) and 1 for heat-related ED visits since these occur almost exclusively in summer. For mortality, constant ‘A’ was defined as the product of the annual-to-daily conversion and the annual-to-warm season conversion: 1/365 * 0.39.
    2. Age range and genders were selected.
    3. Incidence Dataset is set to the set holding the corresponding incidence data—mortality, all-cause ED visits, or heat-related ED visits.
  1. Health functions must be created for each age group, where:
     1. Start Age and End Age are defined accordingly
     2. Function is defined as: DELTAQ*Incidence*POP*A.

In BenMAP, the variable DELTAQ refers to the difference between ‘baseline’ and ‘control’ pollutant data

- - 1. Baseline function is defined as Incidence*POP. This estimates the baseline health effect rate.

1. Define the population dataset under 'Estimate Health Impacts'.
   1. Census 2007 population data should be selected for the historical analysis
   2. ICLUS 2050 population data (loaded in step 4) should be selected for the projected analysis
2. Define the health impact functions to be used for the analysis under 'Estimate Health Impacts'.
3. View and output the results under ‘Health Impact Results’.
